# Membrane bridges and nanodomain partitioning govern membrane protein targeting to lipid droplets

**DOI:** 10.1038/s41556-026-01963-3

**Published:** 2026-05-26

**Authors:** Arda Mizrak, Jacob Kæstel-Hansen, Jessica Matthias, J. Wade Harper, Nikos S. Hatzakis, Robert V. Farese, Tobias C. Walther

**Affiliations:** 1 Department of Cell Biology, Harvard Medical School, Boston, MA USA; 2 Department of Chemistry and Nanoscience Center, University of Copenhagen, Copenhagen, Denmark; 3 Abberior Instruments America, Bethesda, MD USA; 4 Novo Nordisk Center for Optimized Oligo Escape and Control of Disease, University of Copenhagen, Copenhagen, Denmark; 5 Cell Biology Program, Sloan Kettering Institute, Memorial Sloan Kettering Cancer Center, New York, NY USA; 6 Howard Hughes Medical Institute, New York, NY USA; 7 Present Address: Department of Biological Engineering, Massachusetts Institute of Technology, Cambridge, MA USA

**Keywords:** Organelles, Membrane trafficking, Single-molecule biophysics

## Abstract

Numerous metabolic enzymes translocate from the endoplasmic reticulum (ER) membrane bilayer to the lipid droplet (LD) monolayer, where they perform essential functions. Mislocalization of certain LD-targeted membrane proteins, including HSD17B13 and PNPLA3, is implicated in metabolic dysfunction-associated steatotic liver disease. However, the mechanisms governing the trafficking and accumulation of ER proteins on LDs remain poorly understood. Here using minimal fluorescence photon fluxes nanoscopy and highly inclined and laminated optical single-molecule tracking combined with machine learning, we show that HSD17B13, GPAT4 and the model cargo ‘LiveDrop’ diffuse at comparable speeds in the ER and on LDs, but become nano-confined upon reaching the LD surface. Mechanistic dissection of LiveDrop targeting revealed that this confinement, along with protein accumulation on LDs, depends on specific residues within its targeting motif. These residues mediate preferential interactions with nanoscale membrane domains, suggesting that LD-targeted proteins selectively partition into distinct lipid–protein environments that transiently alter local motion and concentrate them at the LD surface. Single-molecule trajectories further revealed bidirectional trafficking of LiveDrop across seipin-containing ER–LD bridges, providing direct evidence for lateral protein transfer across membrane contact sites. These findings establish nanodomain-based confinement as a key mechanism driving selective protein accumulation on LDs and reveal how membrane bridges between organelles facilitate protein sorting.

## Abstract (web summary)

Mizrak et al. use single-molecule tracking to dissect mechanisms of protein targeting to lipid droplets. They show that nanodomain-based confinement drives selective protein accumulation on lipid droplets.

## Main

Lipid droplets (LDs) serve as hubs for cellular metabolism. Unlike membrane-bound organelles, LDs are composed of a hydrophobic core of neutral lipids, primarily triacylglycerols (TGs) and cholesterol esters, surrounded by a phospholipid monolayer rather than a bilayer membrane. This unique architecture supports a specialized proteome rich in enzymes that coordinate lipid synthesis and mobilization. In addition to their established roles in energy storage and membrane production, LD-associated proteins play critical roles in maintaining metabolic homeostasis. Notably, proteins such as PNPLA3 and HSD17B13 have been implicated in the pathogenesis of metabolic disorders, including metabolic dysfunction-associated steatotic liver disease^1–5^.

Numerous LD proteins target to the LD surface from the endoplasmic reticulum (ER) bilayer membrane. These proteins insert into the ER membrane during their synthesis and subsequently traffic to LDs, where they favour localization and accumulation at the monolayer surface. How LD proteins transition between the two organelles in human cells is largely unknown. A current model posits that they traffic to LDs through ER–LD membrane bridges^6–9^. It remains unclear whether individual LDs are connected to the ER through multiple membrane bridges, as observed in *Drosophila* cells^7^, whether the bilayer–monolayer attachment at ER–LD contact sites is structurally stable or how this unique membrane topology influences protein mobility between the two compartments. Also unclear is how ER-derived cargoes accumulate at LDs, rather than equilibrating between the LD and ER. Possible explanations to drive cargo accumulation on LDs include a change in conformation, an LD trapping mechanism or selective ER protein degradation^8,10,11^.

Analysing ER–LD cargo trafficking is challenging because the transition between ER and LDs is extremely rapid, and hence, these events are rare in the lifetime of a protein. In addition, ER to LD trafficking occurs within a dense ER network that maintains contact sites with LDs and other organelles, making events hard to track in space and time. Here, we sought to overcome these challenges in capturing protein targeting between ER and LD by employing single-molecule tracking with minimal fluorescence photon fluxes (MINFLUX) and highly inclined and laminated optical (HILO) fluorescence microscopy.

### Nanodomain interactions alter local mobility on the LD monolayer

**Fig. 1:**
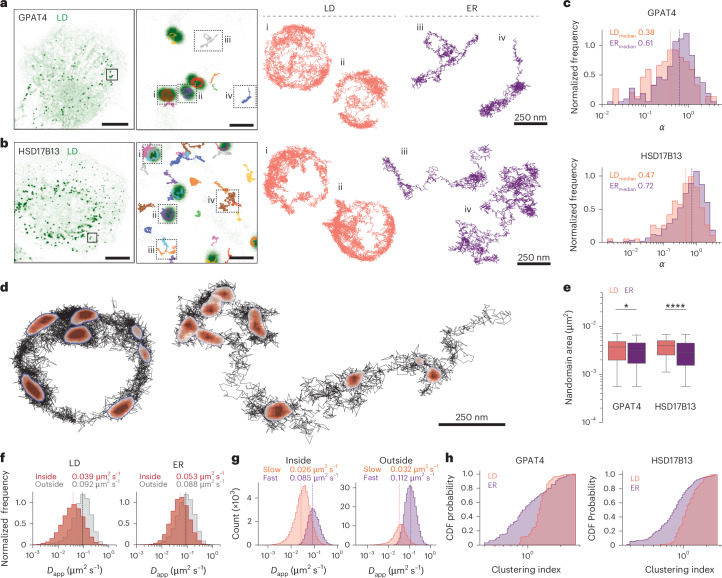
Nanodomain interactions alter molecular motion on LD surface. **a**,**b**, MINFLUX single-molecule trajectories of GPAT4 (**a**) and HSD17B13 (**b**) overlaid on confocal images of cells stained with BODIPY 493/503 to label LDs. Confocal LD images were used to classify trajectories by subcellular localization. Representative examples of single-molecule tracks on LDs and the ER are shown on the right. Scale bars, 10 µm (left), 1 µm (middle) and 250 nm (right). **c**, Histograms of the anomalous diffusion exponent (*α*) for ER-localized (purple) and LD-localized (salmon) tracks of GPAT4 (left) and HSD17B13 (right). *α* values were obtained by fitting a power-law equation to the full MSD curve of each trajectory. Dotted lines indicate medians. 95% confidence intervals: GPAT4_LD_ (0.30 to 0.45), GPAT4_ER_ (0.55 to 0.72), HSD17B13_LD_ (0.38 to 0.60) and HSD17B13_ER_ (0.64, 0.83). A two-sided Mann–Whitney test was used to assess the statistical significance of the difference between the *α* values for the trajectories at each compartment. *P* = 2.3 × 10^−4^ (GPAT4) and *P* = 5.2 × 10^−4^ (HSD17B13). *N*_tracks-GPAT4_ = 149 (LD) and 182 (ER), *N*_tracks-HSD17B13_ = 149 (LD) and 182 (ER). **d**, KDE analysis of GPAT4 single-molecule trajectories on LDs (left) and ER (right). Tracks were segmented into 100 ms intervals, and localization density was computed using a Gaussian kernel. Dark-red contours indicate regions of elevated localization density, representing putative nanodomains on the LD phospholipid monolayer. **e**, Areas of individual nanodomains on the ER and LD membranes were quantified and plotted. The box represents the 25th–75th percentiles (IQR) and the centre line indicates the median. A two-sided Mann–Whitney test was used to assess the statistical significance of the difference in nanodomain sizes at the ER and LD membrane. *P* = 0.015 (GPAT4) and 9.2 × 10^−20^ (HSD17B13). *N*_nanodomains_ = 688 (GPAT4) and 2208 (HSD17B13). **f**, The distribution of the apparent diffusion coefficient (*D*_app_) of GPAT4 within (red) or outside (grey) nanodomains observed on LDs (left) and the ER (right). The *D*_app_ was calculated using a 5-ms rolling window MSD analysis and classified on the basis of the molecule’s position relative to nanodomain boundaries. *N*_steps_ = 306,667 (LD) and 246,591 (ER). A two-sided Mann–Whitney test was used to assess the statistical significance of the difference in *D*_app_ values. *P*_LD_ = 2.6 × 10^−163^, *P*_ER_ = 9.7 × 10^−234^. **g**, The distributions of the fast and slow mobility populations were derived using a Gaussian mixture model from Extended Data Fig. 2a and classified on the basis of whether segments fell inside (left) or outside (right) the nanodomain boundaries. The slow-moving population is predominantly enriched within nanodomains, whereas the fast-moving population is the major species outside of these nano-regions. Median *D*_app_ values of each population are stated on each graph. Mixing weights, *π*_inside_ = 0.68 (slow) and 0.32 (fast), and *π*_outside_ = 0.27 (slow) and 0.73 (fast). *σ*^2^_inside_ = 0.001 (slow) and 0.004 (fast), and *σ*^2^_outside_ = 0.001 (slow) and 0.007 (fast). **h**, The distributions of localization clustering within the nanodomains at the ER and LD membrane for GPAT4 and HSD17B13 were plotted on a cumulative distribution function (CDF) graph. The clustering index was calculated by driving the ratio of mean KDE density at each dense spot and total grid-normalized KDE density. Source data.

To analyse ER–LD membrane protein trafficking, we expressed and sparsely labelled different LD protein cargoes (GPAT4 and HSD17B13) fused to a Halo tag with fluorescent dyes in human SUM159 cells, which display high lipid-storage capacity^12,13^. This allowed for tracking single molecules in live cells using MINFLUX nanoscopy^14,15^ (Fig. 1a,b).

**Extended Data Fig. 1:**
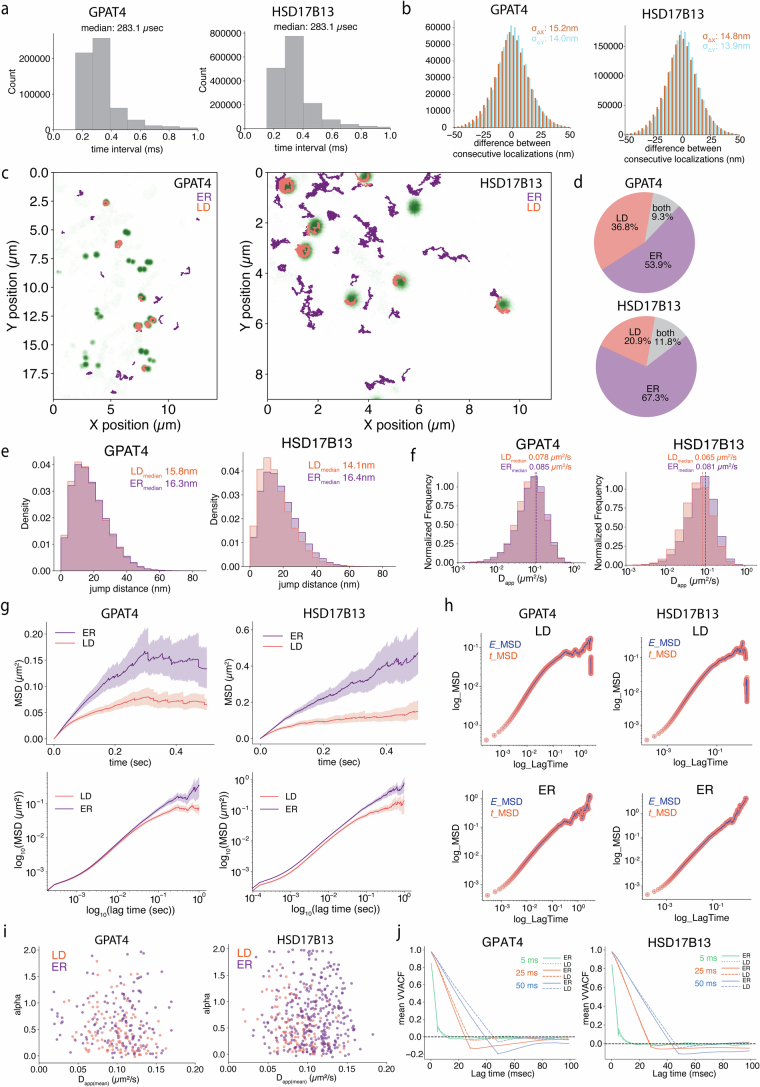
MINFLUX single-molecule analysis of metabolic enzymes GPAT4 and HSD17B13 on the ER and LDs. **a**. The sampling rate of MINFLUX was determined by calculating the time interval between consecutive localizations of individual GPAT4 and HSD17B13 molecules. **b**. The difference between consecutive localizations within single-molecule MINFLUX tracks for GPAT4 (left) and HSD17B13 (right). Standard deviation along each axis was calculated as a proxy for localization precision. **c**. Representative image showing classification of single-molecule tracks based on subcellular localization. Molecules localized within the LD mask were classified as LD-associated (orange), and those outside the mask were assigned to the ER (purple). **d**. Proportion of single-molecule tracks for GPAT4 (left) and HSD17B13 (right) localized to LDs (orange), ER (purple), or spanning both compartments (gray). **e**. Distribution of single-molecule jump distances for GPAT4 (left) and HSD17B13 (right) on the ER (purple) and LD surface (orange). Median values are indicated. **f**. Diffusion coefficients for GPAT4 (left) and HSD17B13 (right), calculated using a 5-ms rolling-window mean squared displacement (MSD) analysis. Values are shown separately for ER-localized (purple) and LD-localized (salmon) trajectories. Dotted lines indicate medians. *N*_steps_ = 551,673 (GPAT4), 1,460,156 (HSD17B13). A two-sided Mann–Whitney test was used to assess the statistical significance of the difference of *D*_app_. *P* = 9.77 × 10^−79^ (GPAT4), 2.1 × 10^−144^ (HSD17B13). **g**. Mean-square displacement (MSD) curves of individual ER- and LD-localized tracks for GPAT4 (left) and HSD17B13 (right), plotted as a function of time interval in linear (top) and log-log scale (bottom). Shaded area represents 95% confidence interval. **h**. Ensemble averaged (E-MSD) and time averaged (t-MSD) were plotted in log-log scale to display the differences in ergodicity for GPAT4 (left) and HSD17B13 (right) at the LD (top) and ER (bottom) membranes. Shaded area represents 95% confidence interval. **i**. 2D correlation map of mean *D*_app_ and alpha values for GPAT4 (left) and HSD17B13 (right). ER and LD localized tracks were colored with purple and salmon, respectively. Mean *D*_app_ values were calculated by taking the average of 5 ms rolling window *D*_app_ values for each track. Pearson’s correlation analysis revealed no significant relationship between α and *D*_app_ (GPAT4: r = −0.09, *P* = 0.15; HSD17B13: r = −0.03, *P* = 0.53). **j**. Velocity–velocity autocorrelation function (VVACF) plots for GPAT4 (left) and HSD17B13 (right). VVACF was calculated by taking the dot product of velocity vectors separated by defined lag times (Δt = 5, 25, and 50 ms) to quantify directional persistence of single-molecule trajectories. Source numerical data are available in source data. Source data

We recorded a total of 2 million single-molecule localizations with 3.5-kHz sampling rate and an estimated <15-nm spatial resolution in the *x*–*y* axes (Extended Data Fig. 1a,b). To analyse the datasets for each cargo, we created a pipeline that first classifies each single-molecule track on the basis of its subcellular location using BODIPY-positive LDs as fiducial markers (Extended Data Fig. 1c,d). We then calculated the apparent diffusion coefficients for each compartment using a 5-ms rolling window mean-squared displacement (MSD). GPAT4’s speed of motion, reflected in the value of the jump distance and its apparent diffusion constant *D*_app_, was comparable after the transition of the molecule from the ER bilayer to the LD monolayer (LD_(median)_ of 0.078 µm^2^ s^−1^ and ER_(median)_ of 0.085 µm^2^ s^−1^) (Extended Data Fig. 1e,f). Similarly, HSD17B13 showed similar mobility on both membranes (Extended Data Fig. 1e,f), suggesting a similar fluidity and crowding of both membranes.

In biological systems, molecular motion frequently deviates from ideal Brownian diffusion due to membrane geometry, organelle-imposed constraints and interactions with binding partners or the local environment. Such effects cause the MSD to depart from a simple linear relationship with time, and this deviation is captured by the anomalous diffusion exponent *α* in the power-law relationship MSD = 4*D*_*α*_ *t*^*α*^, where *α* = 1 corresponds to ideal Brownian motion and deviations from 1 indicate non-Brownian behaviour. Motivated by these considerations, we tested for anomalous diffusion by computing MSD curves across the full duration of single-molecule tracks on the ER or LD membrane.

These full-length MSD curves were fit to the power-law model, and unexpectedly, despite similar *D*_app_ values, we observed clear differences in the temporal organization of molecular motion between the ER and LDs. On LDs, cargoes exhibited lower *α* values, reflecting subdiffusive behaviour in which displacement increases more slowly than expected for free diffusion and instead follows a power-law relationship with time (Extended Data Fig. 1g,f). Correspondingly, the median *α* values for GPAT4 and HSD17B13 were lower on LDs (0.38 and 0.47, respectively) than on the ER (0.61 and 0.72, respectively) (Fig. 1c).

**Extended Data Fig. 2:**
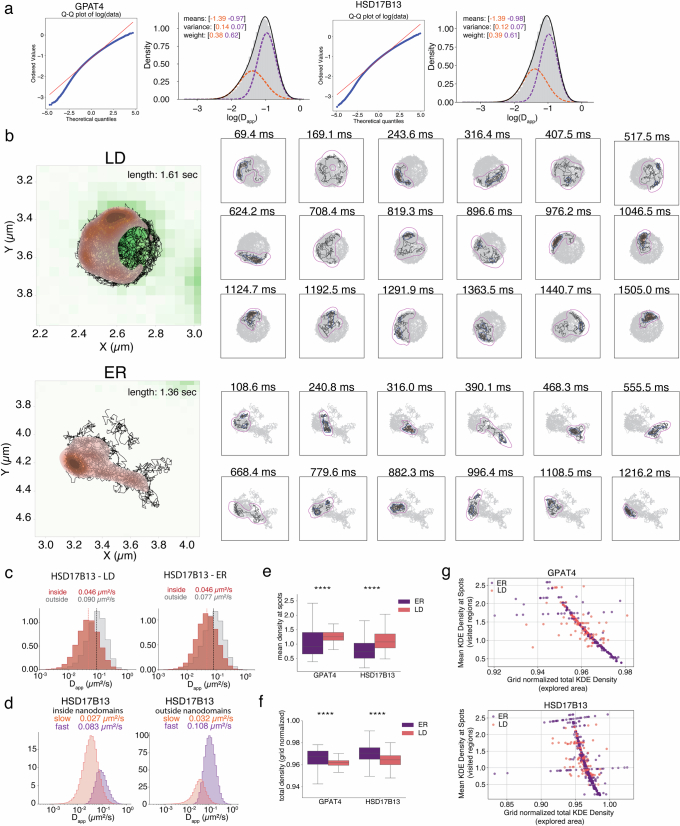
Nanodomain association of model LD cargo analyzed using Gaussian KDE. **a**. A Gaussian mixture model was applied to the *D*_app_ distributions of GPAT4 (left) and HSD17B13 (right) to resolve distinct mobility states within each population. Quantile–quantile (Q–Q) plots and the corresponding *D*_app_ distributions of the fast (purple) and slow (orange) mobility populations are shown. Mixing weights, means and variance are written on the plots. **b**. Representative examples of GPAT4 tracks localized to LDs (top) and the ER (bottom), showing full-track KDE density maps (left). Tracks were segmented into 100 ms windows, and regions exceeding the dataset’s median KDE density were defined as ‘dense.’ The area of these dense regions was quantified. **c**. Apparent diffusion coefficients were calculated for HSD17B13 tracks using a 5-ms rolling window and clustered by whether segments were inside (orange) or outside (purple) nanodomain regions. Median values are indicated by dotted lines. *N*_steps_ = 502,787 (LD), 1,173,290 (ER). A two-sided Mann–Whitney test was used to assess the statistical significance of the difference of *D*_app_ inside and outside the nanodomains. *P* = 4.4 × 10^−215^. **d**. Distributions of the fast and slow mobility populations were derived using a Gaussian mixture model from Extended Data Fig. 2a and classified based on whether segments fell inside (left) or outside (right) the nanodomain boundaries. The slow-moving population is predominantly enriched within nanodomains, whereas the fast-moving population is the major species outside these nano-regions. Median D_app_ values of each population are stated on each graph. Mixing weights π_inside_ = [0.71 (slow), 0.29 (fast)], π_outside_ = [0.27 (slow), 0.73 (fast)]. σ^2^_inside_ = [0.001 (slow), 0.005 (fast)], σ^2^_outside_ = [0.001 (slow), 0.0068 (fast)]. **e**. Mean KDE density within dense regions was computed by dividing the summed KDE values within dense regions by the number of localizations per segment, for ER (purple) and LD (orange) tracks. The box represents the 25th–75th percentiles (IQR), the center line indicates the median. A two-sided unpaired t-tests was used analyze significant differences: *P* = 1.15 × 10^−18^ [*N*_ER_ = 609, *N*_LD_ = 823] for GPAT4, 1.88 × 10^−83^ [*N*_ER_ = 3036, *N*_LD_ = 1332] for HSD17B13. **f**. Total KDE density per segment was normalized to the grid area and compared between ER (purple) and LD (orange) tracks. The box represents the 25th–75th percentiles (IQR), the center line indicates the median. A two-sided unpaired t-tests was used analyze significant differences: *P* = 1.57 × 10^−17^ [*N*_ER_ = 609, *N*_LD_ = 823] for GPAT4, 1.21 × 10⁻^65^ [*N*_ER_ = 3036, *N*_LD_ = 1332] for HSD17B13. **g**. Density enrichment, calculated as the ratio of mean KDE density within dense regions to total normalized KDE density per segment, is shown for ER (purple) and LD (orange) tracks. Source numerical data are available in source data.

Importantly, the differences in anomalous diffusion in the two compartments cannot be explained simply by the smaller surface area or curved geometry of individual LDs compared with the extended ER membrane. Rather, the reduced *α* values on LDs reflect a shift in the local diffusion regime independent of nonergodic trapping or irreversible immobilization (Extended Data Fig. 1h) and occur independently of the overall diffusion speed of individual trajectories (Extended Data Fig. 1i). Velocity–velocity autocorrelation analyses^16^ showed that, particularly at longer timescales, the LD surface imposes long-range constraints on motion and reduce apparent mobility (Extended Data Fig. 1j), indicating that cargoes experience transient, localized constraints on their motion while remaining mobile overall. Consistent with this interpretation, the broad distributions of *D*_app_ observed in both compartments indicate that cargo motion is heterogeneous and comprises multiple mobility states, including slow- and fast-moving populations (Extended Data Fig. 2a). Such heterogeneity supports a model in which nanoscale domains on the LD surface may locally modulate diffusion, leading to nano-confinement events that reduced *α* values in longer timescales (Supplementary Movie 1).

To address this possibility, we mapped the nanodomain distribution of cargoes at the ER and LDs. We temporally segmented individual single-molecule trajectories into 100-ms windows and quantified spatial localization density using Gaussian kernel density estimation (KDE) across ER and LD membranes (Fig. 1d and Extended Data Fig. 2b). We observed nanoscale regions of dense cargo localizations in both compartments, with an average size of 0.0032 ± 0.0016 µm^2^. These nanodomains were present throughout the ER and LD surfaces, but those on LDs were slightly larger on average (Fig. 1e). When cargo entered these nanodomains, its mobility decreased, with *D*_app_ dropping by (on average) 50% (Fig. 1f). Consistent with this reduction, we also observed an increased proportion of molecules in the slow-moving fraction within these domains. (Fig. 1f,g and Extended Data Fig. 2c,d).

Although the nanodomains were observed in both the ER and LD membranes, proteins on LDs were more concentrated within these regions, even though their overall abundance along the track was lower (Extended Data Fig. 2e–g), indicating that cargoes on LDs tend to cluster more tightly into specific spots rather than spreading evenly across the surface (Fig. 1g). These findings suggest that cargo exhibits frequent and spatially concentrated interactions with nanodomains on the LD monolayer, which may contribute to the lower *α* observed at LDs.

### The change in protein behaviour on LDs is mediated by specific amino acid residues

**Fig. 2:**
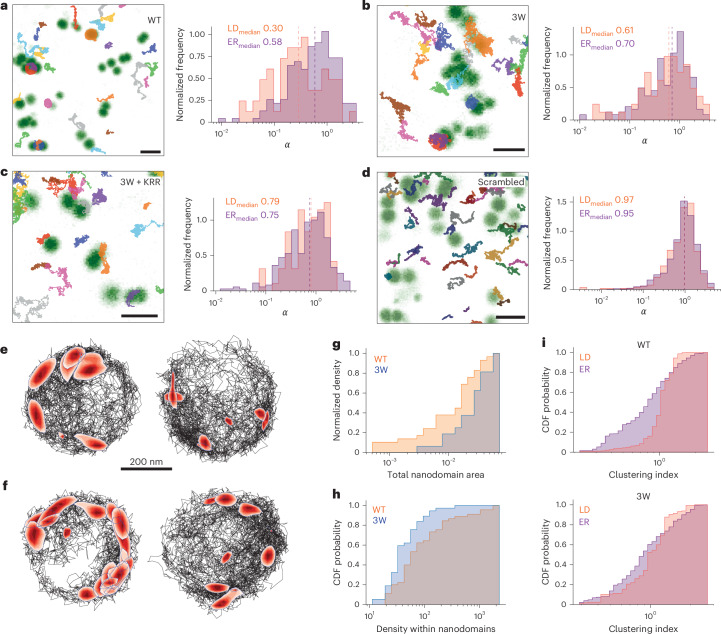
Transient interactions between specific residues and membrane nanodomains drive confinement on LDs. **a**–**d**, MINFLUX single-molecule trajectories and the anomalous exponents (*α*) for LiveDrop WT (**a**), the tryptophan mutant (3W) (**b**), the tryptophan and positively charged mutant (3W + KRR) (**c**) and the scrambled hairpin variant (**d**), overlaid on confocal images of LDs. Scale bars, 1 µm (left) and 250 nm (right). The *α* values for each corresponding trajectory were calculated by fitting a power-law function to the full MSD curve of each trajectory. Distributions are shown for ER-localized (purple) and LD-localized (salmon) tracks. Dotted lines indicate medians. *N*_tracks_ = 285 (WT), 250 (3W), 351 (3W + KRR) and 1,849 (scrambled). A two-sided Mann–Whitney test was used to assess the statistical significance of the difference in *α* values for the ER- and LD-localized tracks. *P*_WT_ = 0.03, *P*_3W_ = 0.83, *P*_3W+KRR_ = 0.52 and *P*_scrambled_ = 0.28. **e**,**f**, The KDE analysis of single-molecule trajectories for WT (**e**) or 3W (**f**). Tracks were segmented into 100-ms intervals, and localization density was computed using a Gaussian kernel. Dark-red contours indicate regions of elevated localization density, representing putative nanodomains. Scale bar, 200 nm. **g**, The cumulative density function probabilities of total nanodomain area per LD in WT (orange) and 3W (blue). WT_median_ = 0.017 µm^2^ and 3W_median_ = 0.032 µm^2^. A two-sided Mann–Whitney test was used to assess the statistical significance of the difference in total nanodomain areas between WT and the 3W mutant. *P* = 0.017. **h**, The cumulative density function probabilities of kernel density of observed localizations for WT (orange) and 3W (blue) within predicted nanodomains. WT_median_ of 67.6 and 3 W_median_ of 36.9. A two-sided Mann–Whitney test was used to assess the statistical significance of the difference in total nanodomain areas between WT and the 3W mutant. *P* = 0.004. **i**, A clustering index was calculated by the ratio of mean KDE density at each dense spot and total grid-normalized KDE density. Cumulative density function probabilities were plotted for WT (left) and 3W (right) trajectories observed on the LD (salmon) and ER (purple) surfaces. A two-sided Mann–Whitney test was used to assess the statistical significance of the difference in *α* values for the ER- and LD-localized tracks. *P*_WT_ = 2.7 × 10^−27^ and *P*_3W_ = 0.008. Source data

**Extended Data Fig. 3:**
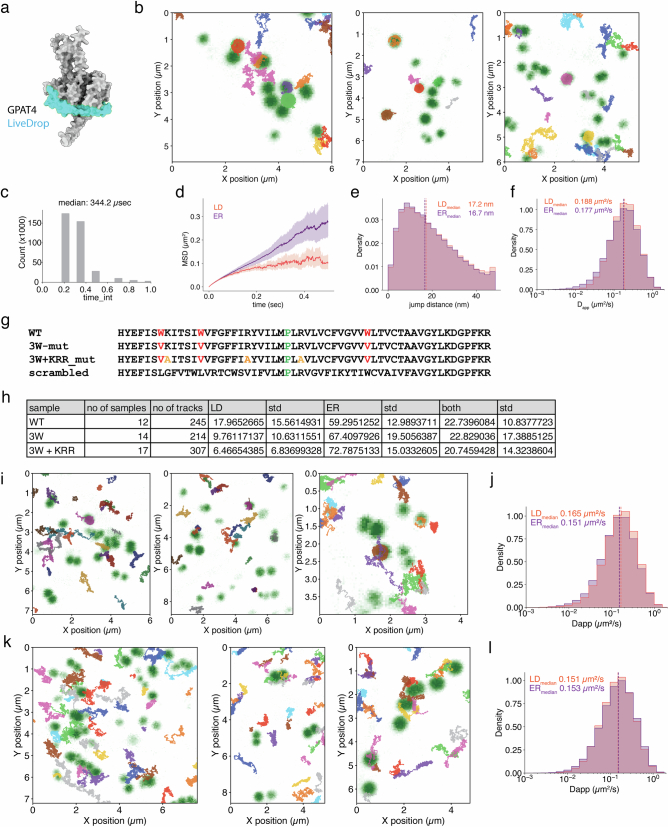
Molecular motion analysis of *LiveDrop* and mutant hairpins using MINFLUX nanoscopy. **a**. AlphaFold-predicted structure of GPAT4, with the engineered hairpin region *LiveDrop* highlighted in blue. **b**. Representative MINFLUX single-molecule tracks of *LiveDrop* in human cells, with individual trajectories shown in different colors. **c**. Sampling rate distribution of MINFLUX tracking for *LiveDrop*. **d**. Mean squared displacement (MSD) curves of *LiveDrop* single-molecule tracks, colored by subcellular location: ER (purple) and LD (orange). Shaded area represents 95% confidence interval. **e**. Jump distance distributions for *LiveDrop* tracks localized to the ER (purple) or LD (orange). Median values are indicated by dotted lines. **f**. Apparent diffusion coefficients were calculated using a 5-ms rolling window for ER- (purple) and LD-localized (orange) *LiveDrop* tracks. A two-sided Mann–Whitney test was used to assess the statistical significance of the difference of *D*_app_. *N*_steps_ = 381,317. *P* = 9.1 × 10^−71^. **g**. Hairpin sequences of *LiveDrop* and its mutants used in this study. The hinge region (green) was preserved to maintain hairpin integrity. **h**. Subcellular distribution of trajectories for each construct, categorized as ER-localized, LD-localized or spanning both compartments. Table shows percentages of tracks found at each compartment. A two sided Welch’s t-test was performed between mutants to assess the significance of changes in localizations. *P*_inside_: 0.13 (WT vs 3 W), 0.03 (WT vs 3 W + KRR), *P*_outside_ 0.21 (WT vs 3 W), 0.01 (WT vs 3 W + KRR), *P*_both_: 0.98 (WT vs 3 W), 0.67 (WT vs 3 W + KRR). **i-l**. Representative MINFLUX tracks and the apparent diffusion coefficients of 3 W mutant tracks (**i**, **j**) and 3 W + KRR (**k, l**). Apparent diffusion coefficients were calculated with a 5-ms rolling window; median values are indicated by dotted lines. A two-sided Mann–Whitney test was used to assess the statistical significance of the difference in *D*_app_. *N*_steps_ = 377,221 (3 W), *P* < 10^−70^ (3 W), *N*_steps_ = 441,999 (3 W + KRR), *P* = 0.002 (3 W + KRR). Source numerical data are available in source data.

LD proteins, such as GPAT4 or HSD17B13, often contain multiple regions that can bind LDs^1,6,17^, complicating the analysis of the underlying mechanisms for membrane protein targeting. To reduce the complexity of the system, we utilized a central hydrophobic hairpin region of GPAT4, known as LiveDrop, which is sufficient to recapitulate ER-to-LD targeting and LD accumulation^6,8^. LiveDrop recapitulated the molecular motions of GPAT4 and HSD17B13, showing similar molecular speeds in both compartments and anomalous diffusion with nano-confinement at LD surfaces (Fig. 2a and Extended Data Fig. 3a–f).

Previous studies showed that the targeting of LiveDrop to LDs relies on tryptophan and positively charged residues^8^. To test whether these residues drive subdiffusive behaviour, we mutated the basic (LiveDrop *‘*KRR’) or bulky hydrophobic (LiveDrop ‘3W’) residues (Extended Data Fig. 3g) and analysed molecular motion with MINFLUX. Mutating tryptophans did not alter LD access, with comparable *D*_app_ values (Extended Data Fig. 3h–j). Moreover, *α* changed only marginally upon LD localization (Fig. 2b), suggesting that these tryptophans contribute to confinement at LDs. More extensive mutations of LiveDrop, such as combining tryptophan and basic residue mutations, essentially abolished LD targeting (Fig. 2c and Extended Data Fig. 3k,l). Instead, we found only rare LD-localized tracks in small ER-adjacent subregions (Fig. 2c). Similarly, a hairpin mutant with the same amino acids scrambled in their sequence (LiveDrop ‘scrambled’) showed no LD localization, and therefore no change in *α* values (Fig. 2d), indicating that both the presence and positioning of tryptophan and charged residues are essential for stable LD localization.

**Extended Data Fig. 4:**
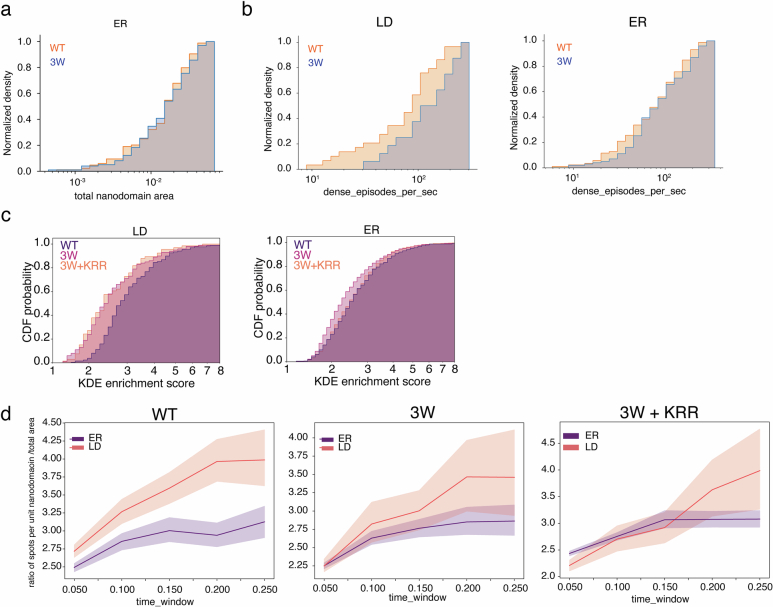
Specific amino acid sequences in the hairpin region mediate nanodomain interaction. **a**. Cumulative density function probabilities of total nanodomain area per LD explored by WT (orange) and 3 W (blue). WT_median_ = 0.018 µm^2^, 3 W_median_ = 0.018 µm^2^. *P* = 0.01. **b**. Normalized nanodomain engagement episodes were plotted as CDF for LD (left) and ER (right) localized trajectories of WT (orange) and 3 W (blue). Normalization was done by dividing number of episodes to track length. Median values for WT_LD_ = 95, WT_ER_ = 85, 3 W_LD_ = 140, 3 W_ER_ = 90. A two-sided Mann–Whitney test was used to assess the statistical significance of the difference in total nanodomain areas between WT and 3 W mutant. *P* = 0.02 (LD), *P* = 0.01 (ER). **c**. KDE-based density enrichment within nanodomains was quantified for WT (blue), 3 W (magenta) and 3W + KRR (orange). Enrichment was defined as the ratio of the mean KDE value at each dense region to the mean KDE of the full grid. Data were plotted as box plots for ER and LD membranes. **d**. Time-resolved density enrichment was plotted for ER-localized (purple) and LD-localized (orange) trajectory segments of WT (left), 3W (middle), and 3W + KRR (right). Segment lengths were varied to assess temporal trends, and enrichment was calculated as described in (**a**) for each time window. Density enrichment was quantified by calculating the ratio of the number of localizations per unit area in dense regions to that in the total KDE area. wild-type (WT) and mutant hairpins. Shaded area represents 95% confidence interval. Source numerical data are available in source data.

To test whether the reduced confinement of the tryptophan mutants reflects altered interactions with LD nanodomains, we compared nanoscale clustering of WT LiveDrop and its mutants. Surprisingly, WT LiveDrop interacted with fewer nanodomains on LDs than the 3W mutant (Fig. 2e–g), but when it entered a nanodomain, it became more tightly concentrated, indicating selective and high-affinity clustering driven by the conserved tryptophans (Fig. 2h). By contrast, the 3W mutant explored a wider set of nanodomains, with an ~80% increase in the total area sampled on LDs, yet failed to show the LD-specific tight clustering characteristic of WT (Fig. 2f–h). On the ER membrane, nanodomain behaviour was similar for WT and 3W, highlighting that WT’s selective clustering is a feature of the LD monolayer (Fig. 2i and Extended Data Fig. 4a). Loss of this selectivity in the 3W mutant led to less dynamic engagement of LD nanodomains and fewer transitions into and out of these regions (Extended Data Fig. 4b). As expected, the 3W + KRR mutant, which rarely reaches LDs, showed minimal localization enrichment within LD nanodomains (Extended Data Fig. 4c,d). Together, these results indicate that conserved tryptophans enable WT LiveDrop to recognize and tightly cluster within LD nanodomains, a feature that underlies its enhanced confinement on the LD surface.

### LD cargo targets all LDs via movement across ER–LD membrane bridges

**Fig. 3:**
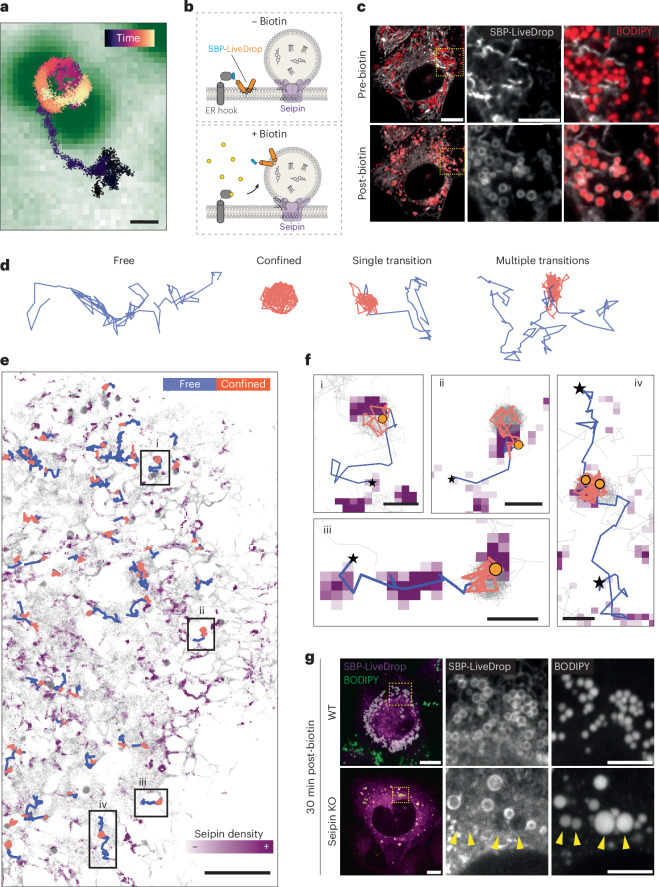
SBP-LiveDrop accesses LDs via seipin-mediated ER–LD contact sites. **a**, Representative MINFLUX single-molecule trajectory of SBP-LiveDrop entering the LD surface. Time progression is colour-coded (start to end, black to yellow). LDs (green) are stained with BODIPY 493/503. Scale bar, 250 nm. **b**, A schematic of synchronized SBP-LiveDrop trafficking from the ER to LDs using the RUSH system. SBP-LiveDrop is retained on the ER membrane through interactions with an ER-localized hook. Biotin addition competes for streptavidin binding, releasing SBP-LiveDrop and enabling real-time monitoring of its trafficking to the LD surface. **c**, Live-cell confocal images of SBP-LiveDrop before and 10 min after biotin-induced release. Cells were pretreated with 250 µM oleic acid overnight to induce LD formation. SBP-LiveDrop was labelled with the JFX554 Halo ligand and released by adding 80 µM biotin. LDs were stained with 1 µM BODIPY 493/503. Scale bars, 10 µm (left) and 5 µm (middle). **d**, The motion types of SBP-LiveDrop classified by DeepSPT. Single-molecule trajectories were categorized into four groups: free-only (blue), confined-only (orange), single transition (free-to-confined or vice versa) and multiple state-switching trajectories. **e**, SBP-LiveDrop single-molecule trajectories plotted over a seipin density map. Cells expressing sparsely labelled SBP-LiveDrop and endogenously tagged seipin were imaged simultaneously using HILO microscopy. An LD prediction algorithm identified trajectories moving from the ER to LDs (Methods). Motion states are colour-coded; other tracks are shown in light grey. Scale bar, 10 µm. **f**, Examples of SBP-LiveDrop trajectories entering LDs through seipin-rich regions. Trajectories are colour-coded by motion state: free (blue) and confined (orange). Stars denote starting positions; orange circles indicate free-to-confined transitions used as proxies for LD entry. Scale bar, 500 nm. **g**, Confocal images of SBP-LiveDrop in WT and seipin KO cells. Cells were incubated overnight with 250 µM oleic acid and imaged by spinning-disc confocal microscopy 30 min after biotin release. Arrowheads indicate LDs in seipin KO cells that failed to recruit SBP-LiveDrop, consistent with a loss of ER–LD membrane contact. Scale bars, 10 µm (left) and 5 µm (right). Source data

We hypothesized that the change in motion behaviour between ER and LD could be used to better understand the route of LiveDrop targeting to LDs, specifically by extracting the coordinates where the protein crossed the bilayer–monolayer membrane contiguity connecting the organelles. Although MINFLUX captured LiveDrop molecules that travel between the two compartments with high resolution (Fig. 3a), because these events are rare, and MINFLUX can only track one molecule at a time, we turned to a different system to comprehensively analyse the trafficking path of LD cargo.

**Extended Data Fig. 5:**
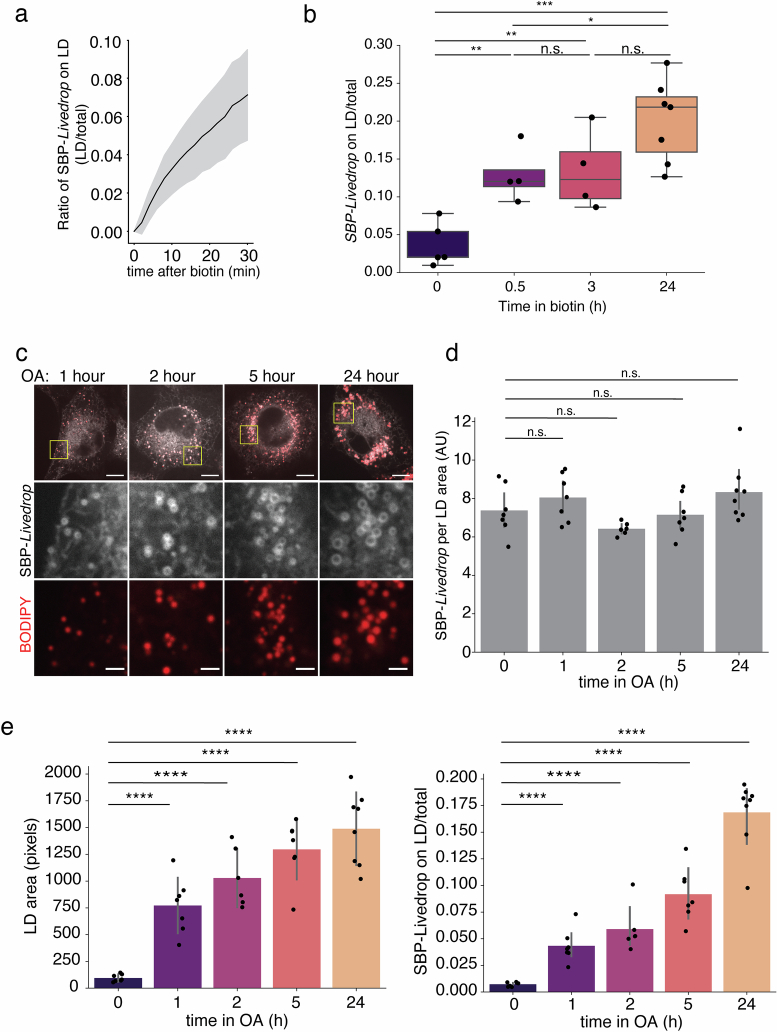
Synchronization of ER-to-LD protein transport using the RUSH system. **a**. Quantification of SBP-*LiveDrop* targeting kinetics after biotin-induced release. Targeting ratio was calculated as the intensity of SBP-*LiveDrop* within the BODIPY-stained LD mask normalized to total cellular SBP-*LiveDrop* intensity and baseline (pre-release) levels. *N*_cells_ = 35. Shaded area represents standard deviation. **b**. SBP-*LiveDrop* targeting ratio to LDs measured at multiple time points post-biotin release and displayed in a boxplot. The box represents the 25th–75th percentiles (IQR), the center line indicates the median. Statistical significance was assessed by a two-sided unpaired t-tests: *P* = 0.004 (0 vs 0.5 h), 0.009 (0 vs 3 h), 0.0001 (0 vs 24 h), 0.86 (0.5 vs 3 h), 0.04 (0.5 vs 24 h), 0.08 (3 vs 24 h). **c**. Representative confocal images showing SBP-*LiveDrop* localization to nascent and mature LDs after biotin release. Cells were pretreated with 250 µM oleic acid for varying durations to induce LD formation, followed by a 30-minute biotin release prior to imaging. LDs were stained with 1 µM BODIPY 493/503. Scale bar: 10 µm (top), 2 µm (bottom). **d**. Quantification of SBP-*LiveDrop* intensity normalized to LD area, calculated by dividing the targeting ratio by the total BODIPY mask area per image, reflecting SBP-*LiveDrop* density on LD surfaces after 30 minutes of biotin release. Time 0 represents no OA addition. Error bars show standard deviation. Values for each field of view (*N* = 7) are overlaid on graphs. Statistical significance was assessed by unpaired t-tests with p-values indicated for comparisons between time points in OA. *P* = 0.33, *N*_cells_ = 23 (0 vs 1 h); *P* = 0.11, *N*_cells_ = 29 (0 vs 2 h); *P* = 0.73, *N*_cells_ = 26 (0 vs 5 h); *P* = 0.21, *N*_cells_ = 32 (0 vs 24 h). **e**. Total LD area (left) and SBP-*LiveDrop* targeting ratio (right) quantified after 30 minutes of biotin release in cells incubated with 250 µM oleic acid for increasing durations. Time 0 represents no OA addition. Error bars show standard deviation. Values for each field of view (*N* = 7) are overlaid on graphs. Statistical significance was assessed by unpaired t-tests with p-values indicated for comparisons between time points in OA. *P* = 0.000052, *N*_cells_ = 23 (0 vs 1 h); *P* = 0.000048, *N*_cells_ = 29 (0 vs 2 h); *P* = 1.3 × 10^−6^, *N*_cells_ = 26 (0 vs 5 h); *P* = 5.9 × 10^−9^, *N*_cells_ = 32 (0 vs 24 h). Source numerical data are available in source data.

To increase the likelihood of observing the ER to LD transition of molecules, we sought to synchronize trafficking by combining single-molecule tracking with an LD-RUSH (Retention Using Selective Hooks) system^18^ (Fig. 3b). We co-expressed an ER hook and LiveDrop fused to a streptavidin-binding peptide (SBP*-*LiveDrop), which retained LiveDrop at the ER membrane even after treatment of cells with oleic acid-containing medium overnight. Subsequent addition of biotin to the culture medium released SBP*-*LiveDrop from the anchor, enabling visualization of ER-to-LD protein trafficking (Fig. 3b,c). Within 10 min of release, LiveDrop relocalized from the ER to essentially all LDs in the cell, where it continued to accumulate over the next 60 min (Extended Data Fig. 5a,b). After biotin release, SBP-LiveDrop localized to LDs irrespective of their size or time of formation, whether nascent or mature (Extended Data Fig. 5c–e), indicating a uniform mechanism that facilitates and preserves membrane continuity between the ER and LDs.

**Extended Data Fig. 6:**
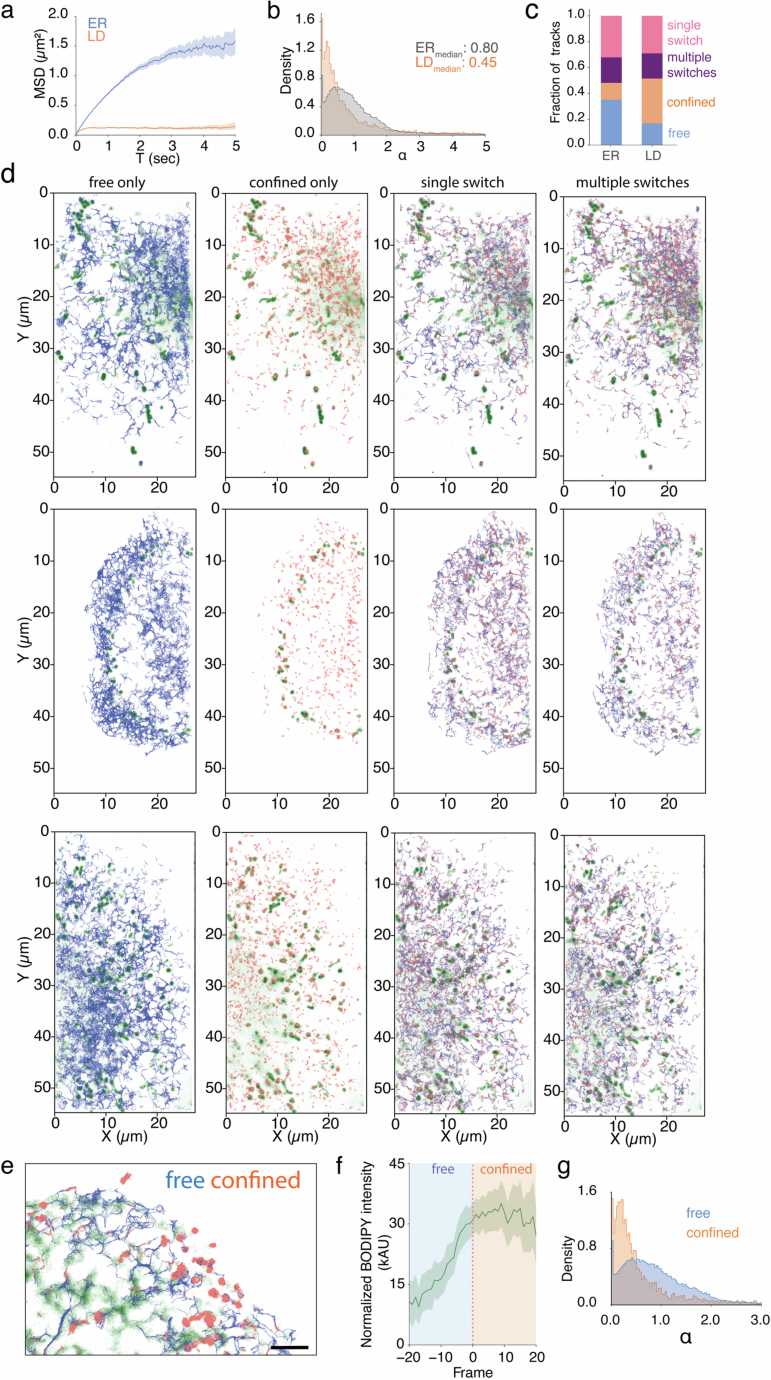
Classification of single-molecule tracks, based on motion states. **a**. Mean-square displacement (MSD) curves of tracks localized to the ER and LD, recorded by HILO imaging. Shaded area represents 95% confidence interval. **b**. Distribution of anomalous diffusion exponent (α) for ER-localized (blue) and LD-localized (orange) tracks. Median α values are indicated. **c**. Representative images showing motion classes of SBP-*LiveDrop* tracks. Tracks were classified using the DeepSPT diffusion pipeline, which analyzes changes in α within individual tracks. Four motion classes are defined: free motion, confined motion, single switch (one transition between free and confined), and multiple switches (multiple transitions). **d**. Representative images showing motion classes of SBP-*LiveDrop* tracks overlaid on ER marker (mEmerald-KDEL). Colocalization analysis shows 96 ± 1.5% of freely diffusive trajectories (*N*_tracks_ = 975) localize to ER regions, with only 4 ± 1.5% observed on LDs. In contrast, 70 ± 1.2% of confined trajectories (*N*_tracks_ = 502) localize to LDs, whereas 29 ± 1.2% colocalize with the ER. **e**. Mean BODIPY intensity along ER-to-LD tracks, aligned to the frame of motion-state switch (frame 0), with standard deviation shown. The increase in BODIPY signal after frame 0 indicates protein entry onto the LD surface. *N* = 219. Shaded area represents 95% confidence interval. **f**. Distribution of α values for tracks classified as free (blue) and confined (orange). Source numerical data are available in source data.

We recorded ~56,000 SBP*-*LiveDrop single-molecule trajectories of sparsely labelled SBP-LiveDrop by using HILO sheet microscopy at various times after biotin release (Supplementary Movies 2 and 3). To categorize single-molecule tracks on the basis of their localization and anomalous diffusion behaviour, we implemented DeepSPT, a machine-learning-based diffusion analysis pipeline^19^ (Fig. 3d and Methods). In agreement with MINFLUX measurements, the fitting of HILO tracks showed a lower *α* exponent on LDs than at the ER, indicating nano-confinement on the monolayer surface (Extended Data Fig. 6a,b). This analysis revealed that tracks moving from the ER to LDs changed behaviour from free to nano-confined motion, with 96% ± 1.5% of freely diffusive trajectories located at the ER, while 70% ± 1.2% of confined trajectories were captured on LDs, enabling temporal classification of ER and LD trajectories (Extended Data Fig. 6c–g).

**Extended Data Fig. 7:**
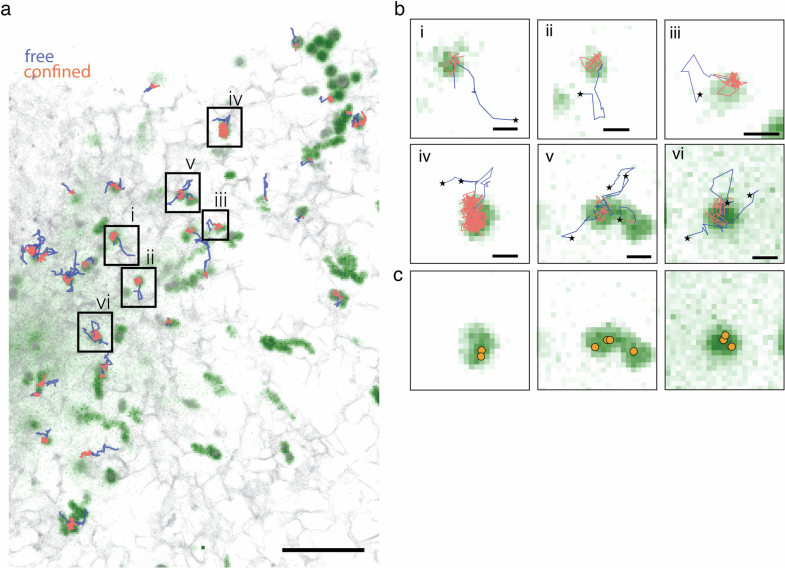
Protein entry onto the LD surface through ER-LD contact sites. **a**. Single-molecule tracks of SBP-*LiveDrop* 8 minutes after biotin release. Cells were treated with 250 µM oleic acid for 3 hours prior to release and labeled with 80 pM JFX554 for HILO imaging. Tracks transition from free motion (blue) to confined motion (orange) as proteins move from the ER membrane to the LD surface. All recorded tracks are shown in light grey. LDs are labeled with BODIPY 493/503 (green). Scale bar: 10 µm. **b**. Representative ER-to-LD trajectories, colored by motion state (blue: free, orange: confined); track start points are marked with stars. Scale bar: 500 nm. **c**. Overlay of coordinates where tracks switch from free to confined motion (from panel **b**) plotted over the LD channel (green).

With DeepSPT, we also captured a few instances of multiple different tracks appearing to access LDs from different directions within a short time (Extended Data Fig. 7a). To test whether one or multiple ER–LD membrane bridges allow protein trafficking, we analysed the coordinates where the switch from ER to LD occurs. These molecules entered BODIPY-stained LDs at similar subregions (Extended Data Fig. 7b,c), suggesting that each of these molecules accesses the LD through the same entry site.

### LiveDrop cargo protein accesses LDs via seipin-mediated membrane bridges

**Extended Data Fig. 8:**
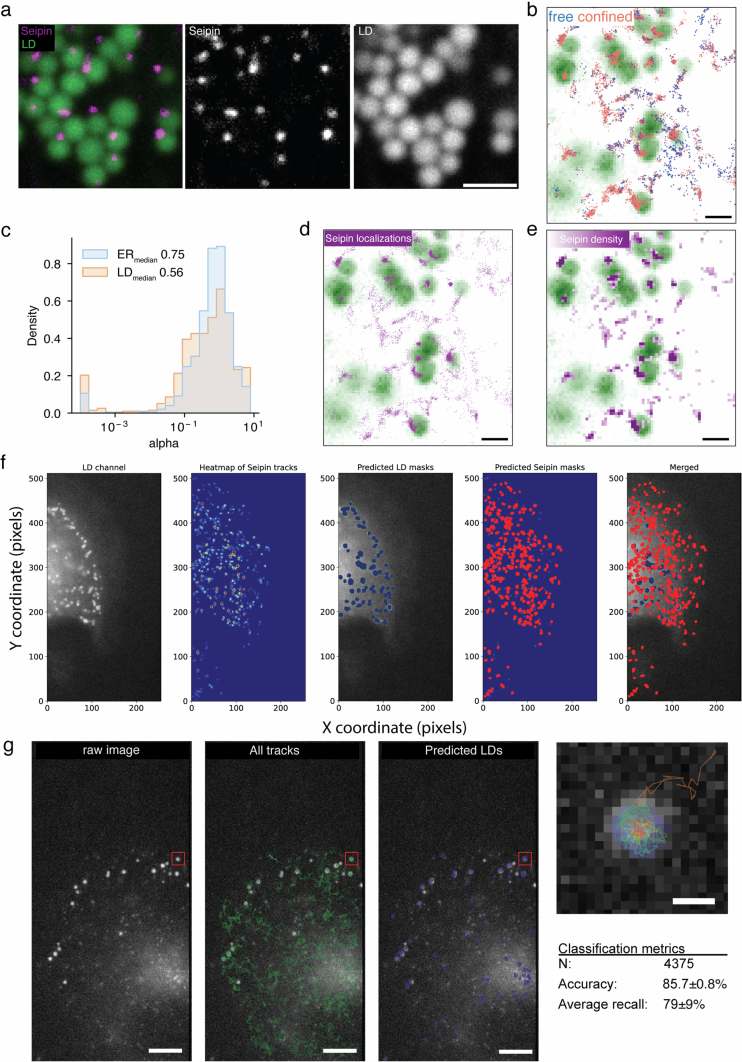
Spatial dynamics of endogenous Seipin at ER-LD contact sites. **a**. Representative spinning disk confocal images of endogenous Seipin-sfGFP in cells incubated overnight with 250 µM oleic acid and stained with LipidTOX Deep Red to label LDs. Scale bar: 2 µm. **b**. Single-molecule localizations of endogenously tagged Seipin, colored by motion class. Cells were transiently transfected with Halo-tagged Plin3 to label LDs, incubated overnight with 250 µM oleic acid, and Plin3 was labeled with JF554X prior to imaging. **c**. Distribution of the anomalous diffusion exponent (α) for Seipin tracks localized to the ER (blue) and LDs (orange). Median α values are indicated. Statistical significance of differences in *D*_*app*_ between ER and LDs was assessed using a two-sided unpaired Mann–Whitney test. *P* = 0.00014. **d**. Spatial plotting of Seipin single-molecule positions from individual complexes over the LD channel, showing enrichment of Seipin near LDs. Scale bar: 2 µm. **e**. Positional density map of Seipin localizations marking ER-LD contact sites. Density is calculated as the frequency of Seipin detections per pixel and represented as a colormap; darker magenta indicates higher density (25+ positions). Scale bar: 2 µm. **f**. LD mask prediction using motion characteristics of *SBP-LiveDrop* tracks. From left to right: HILO images of LD labelled with BODIPY. Heatmap of seipin localization density. HILO images of LD labelled with BODIPY overlaid with LD segmentation. Heatmap of seipin with identified seipin hotspots overlaid. HILO images of LD labelled with BODIPY overlaid with LD mask segmentations and seipin hotspots. Each pixel is equal to 108 nm. **g**. HILO images of LD labelled with BODIPY (left). SBP-*LiveDrop* tracks overlaid (middle). Predicted LD mask based on motion characteristics of SBP-*LiveDrop* (right, see Methods). Zoom-in: LD with SBP-*LiveDrop* tracks classified as LD associating overlaid exemplifying the identification of LD using SBP-*LiveDrop* tracks. Table: Classification metrics for the prediction of LD-localized trajectories to be used for identification of LD localization from SBP-*LiveDrop* tracks alone. Metrics are evaluated by Leave-one-out cross-validation by withholding a single SPT movie as test set and utilizing the remaining for training. N signifies the total number of tracks evaluated in the cross-validation scheme. Accuracy and recall quantify the classification performance across the cross-validation. Scale bar = 5 µm (left), 500 nm (right). Source numerical data are available in source data.

**Extended Data Fig. 9:**
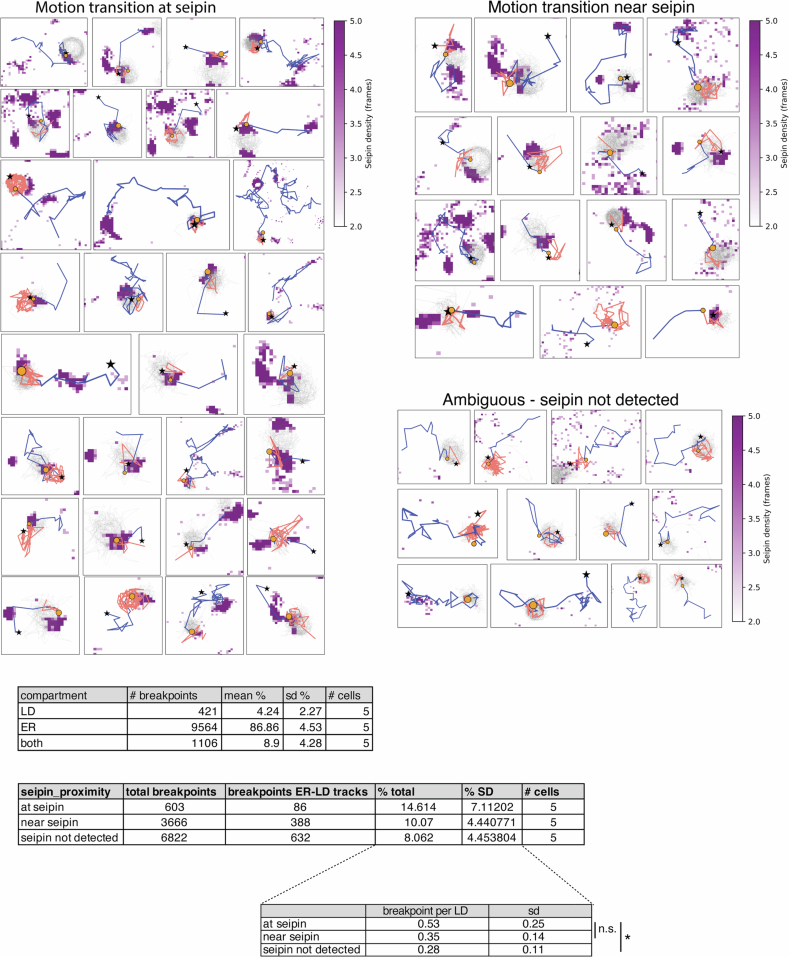
*LiveDrop* trajectories at seipin-enriched ER–LD contact regions. Examples of *LiveDrop* single-molecule trajectories entering or exiting LDs near seipin-enriched regions. Trajectories were filtered by identifying tracks that enter or exit predicted LD masks and undergo a corresponding change in diffusive behavior, from free (blue) to restricted (salmon) motion upon LD entry or from restricted to free motion upon exit, to enrich for bona fide ER–LD transfer events. In some cases, coordinates of the change in motion (orange circles) occur adjacent to, rather than precisely overlapping with, seipin puncta, reflecting limits in spatial assignment of diffusion changepoints and seipin localization. These examples are interpreted as supportive evidence for protein trafficking at seipin-associated ER–LD contact regions. Tracks used to define LD masks are shown in light gray. Each seipin density pixel corresponds to 50 nm. Tables indicate the observed ER-LD trafficking events designated as both compartments that colocalize within or near a seipin containing region as well as the regions where no seipin signal was observed. The values were normalized to the number of lipid droplet like objects for each cell. The final normalized breakpoints per LD represents the likelihood of a diffusion breakpoint at the seipin. A two-sided Welch’s T test was performed to assess the statistical significance of breakpoint enrichment at seipin containing ER-LD contact sites. *P*_at seipin vs near seipin_ = 0.16, *P*_at seipin vs seipin not detected_ = 0.05. Source numerical data are available in source data.

Seipin oligomers are components of the LD assembly complex, forming a single stable focus at ER–LD interfaces^9,12,20,21^ (Extended Data Fig. 8a). To test whether the entry point for SBP*-*LiveDrop to LDs was at seipin oligomers, we simultaneously imaged single SBP*-*LiveDrop molecules and endogenously tagged seipin after biotin release. LD-associated seipin was far less mobile than free seipin, probably owing to its role in maintaining ER–LD membrane contacts^9,12,22,23^ (Extended Data Fig. 8b,c and Supplementary Movie 4). Therefore, seipin’s molecular positions were densely clustered at ER–LD contact sites (Extended Data Fig. 8d,e). DeepSPT analysis of SBP*-*LiveDrop motion characteristics revealed changed motion-track signatures at seipin-dense regions when they travel to LDs (Fig. 3e and Extended Data Fig. 8f,g; Methods). As these tracks transitioned from the ER to LDs, their motion breakpoints were enriched at the focus formed by a stable LD-associated seipin cluster (Fig. 3f and Extended Data Fig. 9), indicating protein movement through a seipin complex.

**Extended Data Fig. 10:**
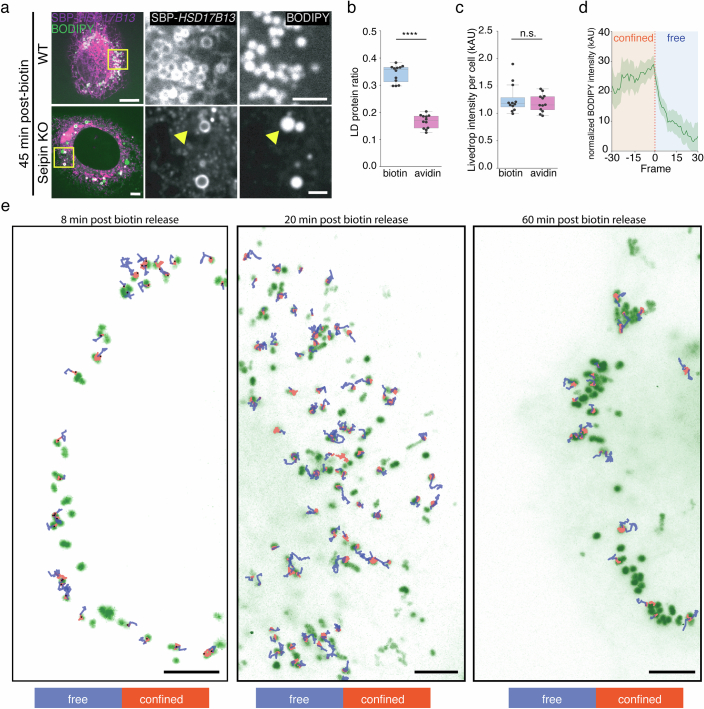
Bidirectional trafficking of *SBP-LiveDrop* single molecules. **a**. Protein partitioning defects in seipin KO cells. The HSD17B13–SBP construct was expressed together with the ER hook in wild-type or seipin-knockout cells. Following overnight oleic acid treatment (200 µM), HSD17B13–SBP was released by addition of 80 µM biotin for 45 min. Consistent with the results shown in Fig. 3g, a subset of lipid droplets in seipin-knockout cells failed to receive protein cargo from the ER after release, indicating a loss of ER–LD membrane contiguity in the absence of seipin. Scale bar = 5 µm (left), 2 µm (magnifications). **b**. Quantification of *SBP-LiveDrop* targeting ratio. The box represents the 25th–75th percentiles (IQR), the center line indicates the median. Values for each field of view (*N* = 12) are overlaid on graphs. A two-sided unpaired t-test was performed to compare biotin and avidin samples. *P* = 5.3 × 10^−10^. *N*_cells_ = 622 (biotin), 632 (avidin). **c**. Quantification of total *SBP-LiveDrop* signal in cells. The box represents the 25th–75th percentiles (IQR), the center line indicates the median. Values for each field of view (*N* = 12) are overlaid on graphs. A two-sided unpaired t-test was performed to compare biotin and avidin samples. *P* = 0.86. *N*_cells_ = 622 (biotin), 632 (avidin). Shaded area represents 95% confidence interval. **d**. BODIPY signal intensity along single-molecule tracks that exited LDs. The switch from confined-to-free motion is indicated as Frame 0. Mean values are shown with standard deviation. *N*_tracks_ = 331. Shaded area represents 95% confidence interval. **e**. Examples of SBP-*LiveDrop* single-molecule tracks entering and exiting LDs at different stages of biotin release. Tracks are colored, based on motion properties. Blue: free; orange: confined. LDs are labeled with BODIPY (green). Scale bar = 5 µm. Source numerical data are available in source data.

Seipin-deficiency leads to defects in LD formation and morphology and LDs detaching from the ER membrane^9,12^. Examining the behaviour of SBP*-*LiveDrop-RUSH in seipin-knockout (KO) cells, we found that, upon release from the ER, SBP*-*LiveDrop targeted some LDs, whereas other LDs did not receive RUSH cargo after 30 min of biotin release, indicating a loss of bilayer–monolayer membrane bridges between the two compartments (Fig. 3g and Extended Data Fig. 10a). It is unclear how some LDs retain continuity with the ER in the absence of seipin; these LDs may receive LiveDrop via transient ER–LD contacts or by budding of small *LiveDrop*-containing LDs that subsequently fuse with mature LDs.

### Cargo movement across seipin complexes is bidirectional

**Fig. 4:**
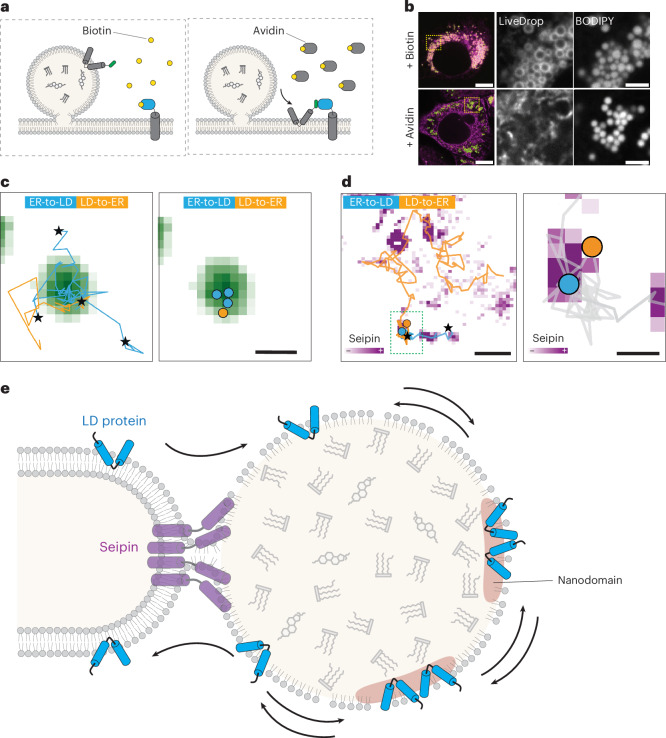
Cargo movement across seipin-mediated ER–LD contact sites is bidirectional. **a**, A schematic of the reverse RUSH assay. Cells were cultured in biotin-containing medium to allow SBP-LiveDrop accumulation on LDs. Subsequent addition of avidin displaced biotin from the ER hook, enabling re-capture of any SBP-LiveDrop molecules that returned to the ER membrane. **b**, Confocal images of SBP-LiveDrop in cells incubated overnight with biotin (top) or after avidin treatment (bottom). Cells were treated with biotin and 250 µM oleic acid overnight to induce LD formation. LD-localized SBP-LiveDrop was labelled with 100 nM JFX554 before avidin addition. After an overnight avidin incubation, cells were imaged using confocal microscopy. LDs were stained with BODIPY 493/503 (green). Scale bars, 10 µm (left) and 2 µm (right). **c**, Representative HILO single-molecule tracks of SBP-LiveDrop exhibiting bidirectional trafficking between the ER and LDs. Left: multiple trajectories showing both entry into and exit from the same LD. Right: spatial coordinates of motion switches are plotted for ER-to-LD (blue) and LD-to-ER (orange) events, overlaid on the LD channel. Stars indicate trajectory start positions. Scale bar, 500 nm. **d**, An example of SBP-LiveDrop bidirectional movement across a seipin-containing ER–LD contact site. Motion switch coordinates in both directions colocalize with a seipin density hotspots near the LD. Scale bars, 1 µm (left) and 250 nm (right). **e**, A model of nanodomain-mediated protein confinement on ER and LD membranes. Seipin maintains ER–LD membrane continuity and regulates lipid and protein flux between the two organelles, thereby shaping the membrane environments that govern protein partitioning and motion. On the LD monolayer, increased local presentation of triglycerides enhances interactions with conserved tryptophan residues, leading to tighter clustering and stronger confinement.

Whether LD proteins can move back to the ER in human cells has been unclear. Single-molecule tracking enabled us to address this question. Since in wild-type (WT) cells, seipin maintains a membrane bridge between ER and LDs that allows proteins to cross, we tested whether these bridges allow SBP-LiveDrop to move back from LDs to the ER. We performed a reverse RUSH experiment (Fig. 4a) in which we incubated the SBP*-*LiveDrop-RUSH-expressing cells with biotin and oleic acid overnight to stimulate LD production and allow SBP*-*LiveDrop to accumulate at LDs. To allow ER hooks to re-capture the prelabelled SBP*-*LiveDrop molecules that escaped from LDs back to the ER, we added excess avidin into the oleate-containing medium the following day to sequester biotin. After overnight avidin incubation, almost all SBP*-*LiveDrop returned to the ER (Fig. 4b). Cellular levels of SBP-LiveDrop did not change after avidin incubation, suggesting that the change in localization was not due to protein turnover (Extended Data Fig. 10b,c).

In agreement with these results, our single-molecule tracking dataset contained instances of SBP*-*LiveDrop molecules that travelled from LD to the ER, and transitioned from nano-confined to free movement (Fig. 4c and Extended Data Fig. 10d). Such LD-to-ER tracks were present at every time point measured, including ones with a short biotin incubation and later stages of biotin release (Extended Data Fig. 10e). The diffusion breakpoints of the reverse tracks were found at sites of LD entry and colocalized with seipin-dense regions (Fig. 4c,d and Extended Data Fig. 9), indicating bidirectional movement of proteins across these regions.

## Discussion

Here, we combine single-molecule tracking with high temporal and spatial resolution to address longstanding questions of how membrane proteins localize to LDs. Our data support a model in which ER-resident membrane proteins are not simply trafficked to LDs but instead become selectively enriched through nanoscale interactions at the LD surface. Notably, GPAT4, HSD17B13 and LiveDrop exhibit similar diffusion speeds in the ER and LD membranes, yet show increased confinement on LDs, indicating that the LD monolayer imposes spatial constraints that promote gradual protein accumulation.

Consistent with this retention-based model, we show that LiveDrop moves bidirectionally across seipin-containing ER–LD bridges, indicating that protein accumulation on LDs is driven by local retention rather than permanent physical barriers. This expands seipin’s function beyond LD nucleation to maintaining a persistent physical ER–LD connection that enables ongoing lipid and protein exchange. While lateral diffusion across seipin-mediated ER–LD connections may be used by other hairpin-containing proteins, alternative routes of LD access have been described in other systems^24^, such as in *Drosophila* cells, where multiple distinct ER–LD bridges contribute to protein loading^7^. Together, these distinct access routes probably provide additional layers of regulation that fine tune protein localization and enable dynamic, selective enrichment of enzymes on LDs, with important implications for metabolic regulation and organelle specialization. Such strategies may be particularly advantageous in cellular contexts that lack dedicated protein translocation machinery.

The mechanism underlying the interaction of LD proteins with nanodomains is currently uncertain. Our data suggest that tryptophans in the hairpin domain do not simply mediate binding, but fine tune compartment-specific nanodomain interactions. On the ER membrane, where TG exposure is more limited and lipid flux is regulated, similar nanodomains may be present but impose weaker constraints on hairpin mobility. On the LD monolayer, increased local concentrations of accessible TG probably enhances interactions with conserved tryptophan residues, resulting in tighter clustering and stronger confinement within nanodomains (Fig. 4e). Accordingly, tryptophan mutations weaken nanodomain interactions, resulting in broader domain sampling, reduced confinement and higher apparent *α* (Fig. 2). These findings suggest that nanodomain engagement is probably driven by biophysical properties that create local heterogeneity within membranes^25,26^. Tryptophans, thus, dynamically couple molecular motion with lipid composition, enhancing compartmental specificity and driving accumulation on the LD surface. Molecular dynamics simulations further support this model, showing that central tryptophan residues (for example, W172 in *Drosophila* GPAT4) can interact with the ester groups of TGs at LD surfaces^8^. How three-dimensional organelle geometry influences trajectory appearance and nanodomain engagement remains a challenging problem that warrants further investigation.

This mechanism of LD cargo accumulation parallels principles of protein sorting in the secretory pathway, where weak and yet specific interactions between proteins and specific lipids, such as sphingolipids and cholesterol, promote clustering of nanodomains targeted to apical membranes in polarized cells^27–30^. These findings support a model in which lipid–protein nanoclustering functions as a mechanism for selective protein sorting and targeting between cellular components.

## Methods

### Chemicals

Janelia Fluor dyes with HaloTag JFX544 and JFX650^31^ were generous gifts from Luke Lavis (Janelia Research Campus). BODIPY 493/503 (D3922) and HCS LipidTOX Deep Red Neutral Lipid Stain (H34477) were purchased from Thermo Fisher Scientific. AUTOdot (SM1000b) was purchased from Abgent. Avidin (A9275), biotin (B4501), fatty acid-free BSA (A6003) and oleic acid (O1008) were purchased from Sigma-Aldrich.

An oleic acid stock solution was prepared at a stock concentration of 10 mM in 3 mM fatty acid-free BSA–PBS solution. The solution was incubated in 37 °C shaking incubator for at least 1 h to dissolve fatty acids, filtered through a 0.22-µm filter, aliquoted and stored at −20 °C.

For RUSH experiments, a 10 µM avidin stock solution was prepared by dissolving the lyophilized powder in sterile PBS and stored at −80 °C. An 80 mM biotin solution (1,000×) was prepared in DMSO and stored at −20 °C.

### Plasmid construction

All PCRs were performed using Q5 High Fidelity DNA Polymerase (M0491, NEB) and restriction enzymes from New England Biolabs. Full-length GPAT4, HSD17B13, SBP-LiveDrop WT, 3W, 3W + KRR and scrambled constructs were cloned using GeneBlocks (IDT) into a lentiviral backbone under the control of the EF1a promoter. RUSH constructs were generated by amplifying the ER hook sequence from Str-ii vector (65300, Addgene) and the SBP-LiveDrop RUSH construct from a geneBlock (IDT). The inserts were cloned into a pLVX lentiviral expression vector (134665, Addgene) using HiFi Assembly (E5520, NEB).

### Lentiviral production

Lentivirus was produced by transfecting HEK293T with a pLVX lentiviral plasmid containing RUSH constructs and standard packaging vectors using *Trans*IT-LTI Transfection Reagent (MIR 2306, Mirus). Viral particles in the supernatant were harvested 2 days post-transfection and stored at −80 °C.

### Cell culture

SUM159 human breast cancer cells were a kind gift from the laboratory of Tomas Kirchhausen (Harvard Medical School) and maintained in DMEM/F-12 GlutaMAX (10565042, Life Technologies) with 5 μg ml^−1^ insulin (Cell Applications), 1 μg ml^−1^ hydrocortisone (Sigma), 5% FBS (10082147, Thermo Fisher), 50 μg ml^−1^ streptomycin and 50 U ml^−1^ penicillin. Cells were maintained in a 5% CO_2_ incubator at 37 °C and at <70% confluency. To induce TG synthesis, cells were treated with growth medium containing 250 μM oleic acid complexed with fatty acid-free BSA.

### Stable cell-line generation

SUM159 cell lines stably expressing RUSH constructs were generated by lentiviral transduction. In brief, cells were seeded into six-well plates in growth medium 2 days before virus introduction. On the day of transduction, cells were treated with 8 µg ml^−1^ Polybrene (TR-1003) for 5 min and incubated with viral supernatant for 2 days. Cells were given 4 days to express the constructs, followed by population sorting using fluorescence-activated cell sorting on a SONY SH800S sorter. The presence of tags in sorted populations was confirmed by fluorescence microscopy before experiments.

### Live cell imaging

SUM159 cells were plated on 35-mm glass-bottom dishes (MatTek Corp.). Before imaging, growth medium was replaced with phenol-free DMEM/F-12 medium containing 15 mM HEPES pH 7.5, 5 μg ml^−1^ insulin, 1 μg ml^−1^ hydrocortisone, 5% FBS, 50 μg ml^−1^ streptomycin and 50 U ml^−1^ penicillin to prevent background fluorescence. For Halo tag labelling, cells were incubated with growth medium containing 100 nM Halo ligand as indicated, followed by three washes with label-free growth medium. For LD staining, cells were treated with BODIPY 493/503 (D3922, Thermo Fisher Scientific) or HCS LipidTOX Deep Red Neutral Lipid Stain (H34477, Thermo Fisher Scientific) at a 1:1,000 dilution for 1 h before imaging. As indicated, 1 μg ml^−1^ Hoechst 33342 (H3570, Thermo Fisher Scientific) was added to growth medium for labelling nuclei.

Live cell imaging was performed using a Nikon Eclipse Ti inverted microscope equipped with Perfect Focus, a CSU-X1 spinning-disc confocal head (Yokogawa), Zyla 4.2 Plus (Andor) or Orca-FUSION (Hamamatsu) sCMOS cameras, and controlled by NIS-Elements software (Nikon). The microscope was equipped with a live chamber (OkoLabs) to maintain cells at 37 °C and 5% CO_2_. Images were acquired through a 100× Plan Apo 1.40 NA objective (Nikon). Cells were excited with solid state lasers (405, 488, 560 or 640 nm) (Agilent). The channels of multicolour images were acquired sequentially. Imaging conditions and analysis workflows can be found in Supplementary Table 1.

### Single-molecule tracking in live cells with MINFLUX nanoscopy

For single-molecule experiments, SUM159 cells that stably express LD constructs were prepared similarly to above, but with the following modifications. To achieve single-molecule labelling, cells were incubated with 50–80 pM JFX650^31^ for 30 min and washed three times with media. Cells were incubated with growth medium containing 250 µM oleic acid for 3 h to induce LD formation. LDs were labelled with 1 µM BODIPY 493/503 30 min before imaging.

MINFLUX data were recorded on a commercial Abberior Instruments MINFLUX setup (Abberior Instruments GmbH), similar to the one reported by Schmidt et al.^15^. The system was equipped with a 100×/1.4 NA magnification oil immersion lens, a 640-nm continuous-wave laser for exciting JFX650-labelled LiveDrop in both confocal and MINFLUX mode, and a 488-nm pulsed laser to image LDs in confocal mode, with a pixel size of 50 nm. MINFLUX tracking was performed with the standard two-dimensional tracking sequence provided by Abberior Instruments by increasing 640-nm laser power over the subsequent iterations. The single-molecule emission was detected at 653–750 nm, with the pinhole set to 0.83 AU. Before and after the actual MINFLUX tracking measurements, confocal images of the LDs were acquired to serve as a reference for the subcellular context. The Abberior Instruments Inspector software with MINFLUX drivers was used to operate the system.

### MINFLUX track analysis and data visualization

MINFLUX single-molecule localization data were acquired across multiple experimental sessions and processed to extract track coordinates and assign localizations relative to LD structures using a custom python script. Raw.npy files containing *x*, *y* and time values for each track were extracted in Pandas. Spatial coordinates were scaled to micrometres using instrument-specific pixel length (50 nm) and offset parameters retrieved from metadata or auxiliary parameter files. To ensure track fidelity, tracks were filtered to exclude those with fewer than 500 time points, limited spatial dispersion or minimal net displacement. The initial localization of each track was excluded to correct for known positional offsets at track initiation. A secondary filtering step removed tracks of which total displacement is smaller than 100 nm in both axes for the entire track, as these probably represent background dye aggregates.

Confocal images of LDs were processed to generate binary masks by applying an intensity thresholding, followed by morphological dilation (5 pixels) to expand detected regions using OpenCV. Pixel coordinates corresponding to the masked LD areas were extracted, and single-molecule track positions were rescaled and compared with this mask to classify localizations as occurring inside or outside LD masks. Tracks were then grouped into three categories: those entirely inside, entirely outside or exhibiting multiple transitions across the LD boundary. Tracks were further analysed to compute stepwise displacements, localization precision and time intervals between observations. Spatial and temporal characteristics of each track were visualized by overlaying trajectories on LD images and constructing density maps. All processed tracks across replicates were aggregated into a final dataset for downstream analysis. Localization precision was calculated by analysing the standard deviation of the changes between each consecutive localization in both axes.

### Apparent diffusion calculation for single-molecule tracks

To capture local variations in diffusivity, a sliding window analysis was performed to calculate the MSD and *D*_app_ across individual tracks. A window size corresponding to a 5-ms time frame was determined on the basis of the median inter-frame interval and applied to each track using a rolling approach. For each window, time lags and squared displacements relative to the initial position were computed. MSD values were derived incrementally for each lag, and the *D*_app_ was estimated from the slope of the MSD curve using the Brownian relation *D* = MSD/(4*t*). Windows with fewer than two valid lag times were excluded from analysis. The resulting time-resolved diffusion coefficients were annotated with the corresponding spatial coordinates and localization category (inside or outside LDs), enabling dynamic assessment of protein mobility across subcellular compartments.

### Anomalous diffusion and confinement analysis

To characterize anomalous diffusion behaviour, MSD curves were fit to a power-law model of the form MSD(*t*) = 4*D* *t*^*α*^. Data points within the entire duration of individual tracks (up to 5 s) were fit using nonlinear least-squares regression in the SciPy package. Fitted parameters were retained only if they satisfied quality control criteria, including physical bounds on *α* (0 < value < 5). Tracks were then stratified on the basis of localization (inside versus outside LDs), and summary statistics, including means, medians and standard deviations, and *α* were computed. Distributions of fit parameters were visualized using log-scaled histograms, with kernel density overlays for the anomalous exponent, allowing the comparison of diffusion dynamics between subcellular regions. Fit parameters and statistical significance were calculated using Mann–Whitney tests provided by the SciPy module.

The ergodicity of single-molecule tracks were analysed by calculating ensemble-averaged MSD (E-MSD) for each localization category by averaging MSD values across all trajectories at each lag time. Time-averaged MSD (T-MSD) was computed independently for each track, aligned by lag time, and subsequently averaged across tracks to obtain the mean T-MSD. The E-MSD and mean T-MSD were plotted on log–log axes to assess ergodicity by comparing population-averaged and time-averaged diffusion behaviour.

To quantify directional persistence and detect deviations from random diffusion, two-dimensional velocities were computed for each trajectory by coarse-graining displacements over defined frame lags and normalizing by the corresponding time intervals to account for variable acquisition timing. The velocity–velocity autocorrelation function was then calculated for each track by quantifying the normalized dot product between velocity vectors separated by increasing time lags^16^. Velocity–velocity autocorrelation function values were computed across multiple frame lags to probe timescale-dependent directional persistence, and results were aggregated across tracks to obtain mean and standard error estimates for each condition.

### Two-state diffusion classification

The distribution of *D*_app_ values was first examined by log-transforming the data, testing for normality and fitting log-normal and Gaussian mixture models (GMMs) to assess whether diffusion behaviour was better described by multiple subpopulations. Model selection using the Bayesian information criterion indicated that a two-component GMM in log space best captured the data, separating slow and fast diffusion states on the basis of their means, variances and mixture weights. Each rolling diffusion value was then probabilistically assigned to a slow or fast state using posterior probabilities derived from the fitted GMM. To account for temporal continuity within individual tracks, a hidden Markov model was implemented using the GMM-derived emission parameters and a defined transition matrix, and Viterbi decoding was applied to infer the most likely sequence of state transitions along each trajectory.

### Analysis of local density dynamics

To quantify the nanoscale clustering behaviour of SBP-LiveDrop, local molecular density was analysed across 100-ms nonoverlapping segments of ER- and LD-localized single-molecule trajectories. For each segment, a two-dimensional Gaussian kernel density estimate (KDE) was computed from the (*x*, *y*) coordinates of localizations. KDE values were evaluated on a regular grid (60 pixels µm^−1^) centred on the segment’s spatial extent with a ±0.2 µm margin. For each segment, dense regions were identified by thresholding the KDE map, and spatial metrics including dense area, total explored area, spot density and the fraction of localizations within dense regions were computed. To capture spatial persistence over time, a union map was generated by taking the pixel-wise maximum density across all segments of a track, and connected-component analysis was used to quantify the distinct nanodomains visited.

The temporal dynamics of nanodomain association were further quantified by classifying localizations as dense or nondense on the basis of local KDE values and tracking state transitions along each trajectory. Entry and exit events, dense-state dwell episodes and dwell durations were calculated using gap-tolerant criteria to account for brief interruptions. The frequency of dense episodes was normalized to total track duration to enable comparison across trajectories of different lengths. All spatial and temporal features were aggregated at both segment and whole-track levels for subsequent comparisons between ER- and LD-localized trajectories and across protein conditions.

### LD-RUSH assay

To retain hairpin constructs at the ER membrane, cells were incubated with media containing 250 µM oleic acid and 50 nM avidin (21121, Sigma) overnight to induce LD formation and sequester free biotin in the growth medium, respectively. The next day, cells were labelled with the indicated Halo ligands, washed three times with fresh medium containing 50 nM avidin and incubated in media containing 250 µM oleic acid, 1 µM BODIPY 493/503 and 50 nM avidin. Release from the ER was initiated by adding 80 µM of biotin (B4501, Sigma) into the imaging dish dropwise with tubing attached to a syringe to prevent any shift in the imaging plane. Image acquisition was started immediately upon biotin release. For kinetics experiments, images were taken every 2 min unless noted otherwise.

Pulse–chase reverse RUSH experiments were performed with cells grown in medium containing 250 µM oleic acid and 1 µM of biotin overnight to allow SBP-LiveDrop to localize to LDs. The next day, cells were labelled with 100 nM JFX554 Halo ligand for 1 h, washed three times with fresh medium and incubated with oleic acid-containing medium supplemented with PBS or avidin overnight to sequester any remaining biotin and allow ER hooks to capture prelabelled SBP-LiveDrop at the ER. At 1 h before imaging, 1 µM BODIPY 493/503 was added to cells to label LDs, and live cells were imaged with a confocal microscope.

### HILO live-cell single-molecule tracking

Single-molecule imaging was performed using a Nikon Ti motorized inverted microscope equipped with an Agilent MLC 400B Laser unit (405/488/561/635), ImageEM EM-CCD camera (Hamamatsu) fitted with a 100× NA 1.49 Apo Total Internal Reflection Fluorescence (TIRF) objective lens (Nikon) combined with a 1.5× tube lens (image pixel size of 0.107 μm). The angle of illumination was optimized to achieve full depth-of-focus of the objective lens with low signal-to-noise ratio (SNR). Simultaneous dual colour imaging experiments were performed with a dual view mirror setup (Optical Insights) equipped with either a 565LP dichroic mirror in series with 525/50 (left) and 600/50 (right) (for JFX554) Chroma filters or a 610LP dichroic mirror followed by 525/50 (left) and 645/75 (right) (for JFX650) Chroma filters for optimal channel separation. Single-molecule images were captured with EM gain at 200 and contrast gain at 2 with 30-ms exposure time combined with no delay acquisition to achieve a frame rate of 31 fps.

### HILO image quantification and track generation

All acquired images were processed and prepared for figures using Fiji^32^. Confocal images were quantified using CellProfiler^33,34^ software with a custom pipeline, including image enhancement, object identification for LD, mask segmentation and quantification of channel intensities to measure SBP-LiveDrop intensity on LDs.

Single-molecule tracks were generated using the Fiji TrackMate plugin^35,36^. Tracks were generated using LAP tracker with a 0.5-µm search radius for each single-molecule spot combined with gap filling of four frames within a 0.7-µm radius. Initial filtering was performed using a custom Python script to filter out tracks shorter than ten frames. Combined tracks were exported in a csv format and analysed in DeepSPT modules (see below).

### Instance segmentation images to capture LDs

To accurately segment LDs with varying sizes, we utilized the deep-learning framework Stardist^37^, which is pretrained to be a versatile segmentation tool in fluorescence microscopy. In each frame, identified LDs were converted into a binary mask of zeroes for background and a single unique integer for the pixels it occupies. Only LDs of fewer than 150 pixels were considered unless LDs were inseparably close or seipin has been knocked out, promoting LDs, in which case large masks were kept. The unique masks identified per frame were tracked in time using Trackpy’s nearest-neighbour linking, with a search range of 5 or 10 pixels depending on visual evaluation and, thus, resulting in a time-persistent unique identifier per LD. The annotation of pixels to either ER or a time-persistent unique LD identifier enables the evaluation of the cellular localization of each SBP-LiveDrop track with individual LD resolution.

### Deep-learning-assisted analysis of temporal diffusional behaviour

To characterize the heterogeneous motion of SBP-LiveDrop, DeepSPT, a modular deep-learning framework specifically designed for interpreting heterogeneous single-particle trajectories, was employed. DeepSPT consists of three core modules: (1) the temporal segmentation of diffusional behaviour, (2) the extraction of trajectory-level diffusion features and (3) a downstream classifier trained on experimental data to perform system-specific classification tasks.

### Temporal segmentation and quantification of diffusional behaviour

SBP-LiveDrop trajectories were input directly into DeepSPT’s temporal segmentation module, which predicts a diffusional state and associated confidence scores at each time point. Diffusion modes observed on ER and LD membranes are diverse, but generally more restricted on LDs owing to their limited area. To simplify behavioural classification, diffusion states were binned as either ‘Free’ (Brownian or directed motion) or ‘Restricted’ (subdiffusive or confined motion). These frame-wise predictions were used to identify diffusion state transitions and evaluate how these dynamics correlated with cellular localization. In parallel, trajectory-level features were extracted using DeepSPT’s second module, including the diffusion coefficient (estimated from single time interval MSD), the anomalous diffusion exponent (*α*, from MSD = 4*D t*^α^), the explored area and the frequency of free versus restricted states.

### DeepSPT feature representation of SBP-LiveDrop trajectories

Features derived from the first two modules were concatenated into fixed-length trajectory embeddings that included the number and sequence of transitions between free and restricted states, the global diffusion metrics and the averaged features within each state. These representations were then used to train DeepSPT’s downstream classifier to distinguish ER- versus LD-localized tracks. The classifier consisted of an ensemble of three random forest models, each with 100 trees and maximum depths of five, six or seven. Final predictions were made by majority vote. To mitigate class imbalance—owing to most SBP-LiveDrop tracks residing in the ER—random oversampling of LD-classified tracks was performed. Classifier performance was evaluated using leave-one-group-out cross-validation, where each biological replicate (that is, independent HILO experiment) was treated as a separate group, ensuring the model generalized across experimental conditions and cell-to-cell variability.

### Classification of trajectory cellular location solely by diffusion and model validation

DeepSPT’s third module utilizes the feature representation derived from the first two modules to provide classification directly using experimental data. The downstream classifier employed for the prediction of SBP-LiveDrop’s cellular localization consists of an ensemble of three random forest classifiers each consisting of 100 tree estimators and with maximum tree depths of five, six and seven, respectively. The final ensemble prediction is taken as the mode prediction of the three classifiers. To overcome biased predictions due to class imbalance, as most SBP-LiveDrop tracks are on the ER, random oversampling of the minority class was implemented. Model performance was evaluated using leave-one-group-out cross-validation, where each group is a biological replicate (that is, an independent TIRF single-particle-tracking experiment) to confirm the model’s ability to generalize across experimental variability and to unseen cells.

### Identification of LD-associated seipin

To identify LD-associated seipin assemblies, we combined diffusional behaviour classification with spatial density mapping. Tracks classified as >90% free-diffusing by DeepSPT were excluded. The remaining tracks were converted into a two-dimensional positional histogram to generate a seipin density heat map. This heat map was then binarized using a dataset-specific threshold and processed with Laplacian of Gaussian filtering (via scikit-image^38^), using a maximum sigma of 2 and a detection threshold of 0.1, to identify discrete regions of high-density, presumed to be LD-associated seipin foci.

### LD prediction and spatial segmentation of seipin-localized diffusion breakpoints

LD-like structures were segmented by thresholding and morphologically filtering LiveDrop density maps to retain small, approximately circular objects, and independently segmented seipin-enriched ER subdomains from seipin localization density maps. Using these masks, we classified diffusion breakpoints along single-molecule trajectories on the basis of their spatial proximity to seipin (‘at seipin’, ‘near seipin’ or ‘seipin not detected’) and on whether the parent track occupied the ER, LD or both compartments. To account for variability in LD abundance across cells and experiments, we normalized breakpoint counts to the number of LD-like objects per sample, yielding breakpoint rates per LD rather than simple fractions. This analysis revealed a strong enrichment of ER–LD transition-associated breakpoints at seipin-enriched regions, indicating that seipin defines preferred sites where LD-associated proteins undergo state changes during ER–LD trafficking.

### Evaluation of linking error potential

The evaluation of linking error potential was performed by calculating the distance from every individual SBP-LiveDrop detection in frame, *i*, to every detection in frame, *i*+1, and counting the number of detections within the SBP-LiveDrop tracking search range.

### Statistics and reproducibility

Unless otherwise stated, results are presented as the mean ± standard deviation. Statistical analyses of results were performed using the SciPy statistics package in Python. Statistical tests used for analysis are indicated in figure legends. Statistically significant calculations were denoted with * for *P* < 0.05, ** for *P* < 0.01, *** for *P* < 0.001 and **** for *P* < 0.0001. Actual *P* values are included in the figure legends. The number of independent experiments is indicated in the figure legends. All experiments were repeated independently at least twice with similar results. No statistical methods were used to predetermine sample sizes. Blinding was not performed during data acquisition or analysis. For imaging experiments, cells were randomly selected for analysis, and at least three independent measurements were performed per condition.

### Reporting summary

Further information on research design is available in the Nature Portfolio Reporting Summary linked to this article.

## Online content

Any methods, additional references, Nature Portfolio reporting summaries, source data, extended data, supplementary information, acknowledgements, peer review information; details of author contributions and competing interests; and statements of data and code availability are available at 10.1038/s41556-026-01963-3.

## Supplementary information

Supplementary Information Supplementary Movies 1–4.

Reporting Summary

Peer Review file

Supplementary Table 1 Overview of microscope configuration, imaging conditions and analysis workflows used in this study.

Supplementary Video 1 Molecular motion of GPAT4 single molecules on a lipid droplet captured by MINFLUX nanoscopy.

Supplementary Video 2 Single-molecule tracking of SBP-LiveDrop following biotin release.

Supplementary Video 3 Single-molecule tracking of SBP-LiveDrop at the endoplasmic reticulum.

Supplementary Video 4 Interaction dynamics of seipin with lipid droplets.

## Source data

Source Data Fig. 1 Statistical source data.

Source Data Fig. 2 Statistical source data.

Source Data Fig. 3 Statistical source data.

Source Data Extended Data Fig. 1 Statistical source data.

## Data Availability

All raw imaging data for HILO and MINFLUX experiments are available via Mendeley Data at https://data.mendeley.com/datasets/zbtvr59j2p/1. All other data supporting the findings of this study are available from the corresponding author on reasonable request. Source data are provided with this paper.

## Code availability

Code and data analysis for MINFLUX tracking datasets are available via GitHub at https://github.com/amizrak/MINFLUX_analysis.git.
