# Supplementary material for: Membrane bridges and nanodomain partitioning govern membrane protein targeting to lipid droplets
Source: Nat Cell Biol. 2026 May 26;28(6):1235–43. doi: 10.1038/s41556-026-01963-3 (PMC13278972; doi:10.1038/s41556-026-01963-3)
Supplement: Supplementary file 1 — Supplementary Movies 1–4. [file 41556_2026_1963_MOESM1_ESM.pdf]

nature cell biology

Supplementary information

https://doi.org/10.1038/s41556-026-01963-3

Membrane bridges and nanodomain partitioning govern membrane protein targeting to lipid droplets

In the format provided by the authors and unedited

Supplementary Movies 1-4

Supplementary Movie 1 | Molecular motion of GPAT4 single molecules on a lipid droplet captured by MINFLUX nanoscopy. Over 10,000 single-molecule localizations were recorded on a single lipid droplet over 3 s. The video is shown at 8× slower speed to visualize dynamic, bidirectional motion within nanodomains, characterized by transient yet repetitive confinement events.

Supplementary Movie 2 | Single-molecule tracking of SBP-*LiveDrop* following biotin release. Addition of biotin releases sparsely labeled SBP-*LiveDrop*, enabling continuous imaging at 30 Hz under HILO illumination. This allows real-time visualization of SBP-*LiveDrop* (magenta) trafficking from the ER to lipid droplets labeled with BODIPY (green), highlighting protein exchange between membranes. Scale bar, 10 µm.

Supplementary Movie 3 | Single-molecule tracking of SBP-*LiveDrop* at the endoplasmic reticulum. Sparsely labeled SBP-*LiveDrop* molecules (magenta) were imaged continuously at 30 Hz under HILO illumination to assess motion within the ER network, labeled with mEmerald-KDEL (green), revealing diffusive behavior within the continuous membrane system. Scale bar, 10 µm.

Supplementary Movie 4 | Interaction dynamics of seipin with lipid droplets. Endogenously tagged seipin (seipin–sfGFP, green) was imaged continuously at 30 Hz under HILO illumination to capture its dynamic behavior at ER–lipid droplet interfaces. Lipid droplets are labeled in magenta. Seipin exhibits confined motion at ER–LD junctions, consistent with stable residency at membrane bridges. Scale bar, 10 µm.
