# Supplementary material for: Membrane bridges and nanodomain partitioning govern membrane protein targeting to lipid droplets
Source: Nat Cell Biol. 2026 May 26;28(6):1235–43. doi: 10.1038/s41556-026-01963-3 (PMC13278972; doi:10.1038/s41556-026-01963-3)
Supplement: Supplementary file 3 — Peer Review file [file 41556_2026_1963_MOESM3_ESM.pdf]

nature portfolio

# Peer Review File

# Membrane bridges and nanodomain partitioning govern membrane protein targeting to lipid droplets

Corresponding Author: Professor Tobias Walther

Version 0:

Decision Letter:

*Please delete the link to your author homepage if you wish to forward this email to co-authors.

Dear Tobi,

Thank you again for submitting your manuscript, "Organelle bridges and nanodomain partitioning govern targeting of membrane-embedded proteins to lipid droplets", to Nature Cell Biology. It has now been seen by 3 referees, who are experts in super-resolution imaging and single-molecule tracking (Referee #1); protein trafficking (Referee #2); and LDs (Referee #3). As you will see from their comments (attached below), they find this work of potential interest but have raised substantial concerns, which in our view would need to be addressed with considerable revisions before we can consider publication in Nature Cell Biology.

As per our standard process, we have now discussed the referee reports in detail within the editorial team, including the chief editor, to identify key referee points that should be addressed with priority, and requests that are overruled as being beyond the scope of the current study. To guide the scope of the revisions, I have listed these points below. As you know, our standard revision period is about 6 months and we are committed to providing a fair and constructive peer-review process, so please feel free to contact me if you would like to discuss any of the referee comments further or if you anticipate any issues or delays in revision.

I should stress that the referees' concerns regarding the analyses and strength of the biological conclusions will need to be addressed experimentally thoroughly, and reconsideration of the study for this journal and re-engagement of the referees will depend on the strength of these revisions. We recommend dedicating efforts in revision to address the reviews as follows:

(i) Rev#1 had concerns about the data interpretation and analyses that need to be addressed for reconsideration at the journal: Rev#1 bullets number #1, 2, 3, 4, 5, 6, 7, 8, 9, 10, 11, 12, 13, 14, 15

(ii) All reviewers asked for analyses to strengthen the model of transport of cargo protein between ER and LDs and to address the role of seipin. Please address their concerns rigorously:

Rev#1 bullet #17; Rev#2 experimental queries, bullets #1-2; Rev#3 points #2-3-4-5

(iii) All reviewers asked for additional simulations or rationale supporting the proposed change in LiveDrop conformation shown in figure 4 and we agree that this needs to be addressed (Rev#1 bullet #16; Rev#2 last bulleted experimental query; Rev#3 point #6)

(iv) Lastly, please address all other minor or technical points, requests for clarification or additional discussion or text edits, and Rev#2's conceptual points.

(v) Finally, please pay close attention to our guidelines on statistical and methodological reporting (listed below), as failure to do so may delay the reconsideration of the revised manuscript. In particular, please provide:

- a Supplementary Figure including unprocessed images of all gels/blots in the form of a multi-page pdf file. Please ensure that blots/gels are labeled and the sections presented in the figures are clearly indicated.

- a Supplementary Table including all numerical source data in Excel format, with data for different figures provided as different sheets within a single Excel file. The file should include source data giving rise to graphical representations and statistical descriptions in the paper and for all instances where the figures present representative experiments of multiple independent repeats, the source data of all repeats should be provided.

We would be happy to consider a revised manuscript that would satisfactorily address these points, unless a similar paper is published elsewhere or is accepted for publication in Nature Cell Biology in the meantime.

When revising the manuscript please:

- ensure that it conforms to our format instructions and publication policies (see below and https://www.nature.com/nature/for-authors).

- provide a point-by-point rebuttal to the full referee reports verbatim, as provided at the end of this letter.

- for any revision that includes light microscopy data, we ask our authors to please include a completed light microscopy reporting table https://www.nature.com/documents/Light_microscopy_reporting_table.xlsx to ensure the methods are described thoroughly. The table will be available to reviewers and ultimately published should the manuscript be accepted at the journal.

- provide the completed Reporting Summary (found here <a href="https://www.nature.com/documents/nr-reporting-summary.pdf">https://www.nature.com/documents/nr-reporting-summary.pdf</a>). This is essential for reconsideration of the manuscript will be available to editors and referees in the event of peer review. For more information see <a href="http://www.nature.com/authors/policies/availability.html">http://www.nature.com/authors/policies/availability.html</a> or contact me.

When submitting the revised version of your manuscript, please pay close attention to our href="https://www.nature.com/nature-portfolio/editorial-policies/image-integrity">Digital Image Integrity Guidelines.</a> and to the following points below:

-- that unprocessed scans are clearly labelled and match the gels and western blots presented in figures.
-- that control panels for gels and western blots are appropriately described as loading on sample processing controls
-- all images in the paper are checked for duplication of panels and for splicing of gel lanes.

Finally, please ensure that you retain unprocessed data and metadata files after publication, ideally archiving data in perpetuity, as these may be requested during the peer review and production process or after publication if any issues arise.

Nature Cell Biology is committed to improving transparency in authorship. As part of our efforts in this direction, we are now requesting that all authors identified as ‘corresponding author’ on published papers create and link their Open Researcher and Contributor Identifier (ORCID) with their account on the Manuscript Tracking System (MTS), prior to acceptance. ORCID helps the scientific community achieve unambiguous attribution of all scholarly contributions. You can create and link your ORCID from the home page of the MTS by clicking on ‘Modify my Springer Nature account’. For more information please visit please visit <a href="http://www.springernature.com/orcid">www.springernature.com/orcid</a>.

This journal strongly supports public availability of data. Please place the data used in your paper into a public data repository, or alternatively, present the data as Supplementary Information. If data can only be shared on request, please explain why in your Data Availability Statement, and also in the correspondence with your editor. Please note that for some data types, deposition in a public repository is mandatory - more information on our data deposition policies and available repositories appears below.

Please submit the revised manuscript files and the point-by-point rebuttal to the referee comments using this link:

Link Redacted

*This url links to your confidential home page and associated information about manuscripts you may have submitted or be reviewing for us. If you wish to forward this email to co-authors, please delete the link to your homepage.

We hope that you will find our referees' comments and editorial guidance helpful. Please do not hesitate to contact me if there is anything you would like to discuss. Thank you again for considering NCB for your work.

Best wishes,

Melina

Melina Casadio, PhD
Senior Editor, Nature Cell Biology
Consulting Editor, Nature Structural & Molecular Biology
ORCID ID: https://orcid.org/0000-0003-2389-2243

Reviewers' Comments:

Reviewer #1 (Remarks to the Author):

Mizrak et al study the dynamics of membrane proteins that shuttle between the ER and lipid droplets (LDs). They employ cutting-edge microscopy (primarily MINFLUX) to follow single proteins in the cells. They recognize confinement in nanodomains on LDs and bridge crossing between the ER and LDs as mechanisms of prime interest. Despite these findings and advanced imaging technique, I have major reservations regarding how the data is analyzed and interpreted:

•Fig. 1 – The authors should first show the entire cell.
•Highlights of the ER are expected, while shown only for LDs
•The distributions of D and alpha are very broad for both proteins under study. Why? Can the authors segment the trajectories to distinguish different mobility characteristics within single trajectories?
•Is there s correlation between D and alpha values? Show a 2D map of these parameters for individual trajectories.
•A main concern relates to the definition of the nanodomains. Could it be that there is excluded volume on the LDs? How does its 3D shape affect the appearance of the trajectories? Can the authors track molecules in 3D or provide 3D cell images, as they employ spinning disk confocal imaging in the study?
•Panel f – what are the associated errors in C and D? Are the differences in D values significant?
•Extended Fig. 1, panel f – How do the graphs look like using log-log representation?
•Are the different random walks ergodic under the various conditions? The authors should conduct both time and ensemble averaging of MSDs and compare them.
•The authors are advised to employ velocity-velocity autocorrelation to further inspect differences in mobility mechanisms.
•Fig. 2 – It is hard to evaluate and thus unclear whether the protein mutations have a significant effect, as no errors are shown for panels b,e,h,k. Overall, these effects seem quite modest.
•How were graphs in panels c,f,I,l normalized?
•Fig. 3 – A distinct and independent marker for ER is expected, not just for LDs.
•What are the potential issues of cell treatment with oleic acids?
•No statistics are shown
•Fig. 4 – No statistics are shown.
•The scheme in Panel e is very speculative. No evidence is provided for the conformational and orientation changes.
•ED Fig. 8a shows seipin clusters. Could trapping seipin in such protein clusters be the main mechanism of confinement? How can this mechanism be excluded?

Reviewer #2 (Remarks to the Author):

The manuscript by Mizrak et al. represents a technical tour de force that clarifies several fundamental aspects of protein targeting to lipid droplets (LDs) in mammalian cells. The central advance is the direct, quantitative characterization of ER-to-LD partitioning in live cells using a model cargo, "live drop." Live drop consists of the hairpin membrane anchor of GPAT4, a lipid enzyme that dynamically partitions between the ER and LDs. Coupled with automated, machine learning–based trace analysis, this work provides, to my knowledge, the first unbiased quantitative investigation of protein targeting to LDs in live cells.
Key findings include the demonstration that the membrane anchors of GPAT4, HSD17B13, and live drop govern ER–LD partitioning and form previously unrecognized nanodomains on the LD surface that mediate LD trapping. The data further establish that these membrane proteins access LDs from the ER through seipin-marked contact sites, which appear to enable bidirectional transport.
The data and figures are of exceptional quality, and the conclusions are clearly presented. This study has the potential to become a "classic" in the LD field and beyond. I strongly support publication in Nature Cell Biology, contingent upon revision of the following points:

Experimental queries:
• The study introduces full-length GPAT4, HSD17B13, and PNPLA3 as cargoes of interest, but does not test whether seipin loss alters their partitioning. After the live drop analysis, it would be important to characterize at least one of these full-length cargoes in seipin knockout cells.
• Alternatively, it would be informative to assess whether KRR or WW mutations exhibit defects in nanodomain trapping similar to live drop. Would such results help explain the LD phenotypes observed in seipin loss-of-function models?
• The conformational changes of live drop presented in Figure 4 require clarification. The proposed model contradicts Olarte et al. (2020, Dev Cell), which described the hairpin as remaining "closed." Could the authors provide additional simulations or rationale supporting their interpretation?

Conceptual comments
• The Walther/Farese group previously introduced GPAT4 as a late ER-to-LD cargo that targets LDs through seipin-independent bridges (e.g., Song et al., 2022 NCB; Olarte et al., 2022 Trends Biochem Sci; Wilfling et al., 2013 Dev Cell). This manuscript now suggests GPAT4 enters LDs via seipin-bridges. The mixing between "bridges" and "seipin contact sites" may confuse readers, as these have previously been explicitly distinguished. The authors should clarify whether this reflects cell-type specificity (e.g., flies versus mammals) or whether live drop behaves differently because it contains only a single LD-targeting domain.

• The title “Organelle bridges…” is somewhat misleading, as this study focuses exclusively on ER–LD contact sites. A more precise title, such as “ER contact sites and nanodomain partitioning…”, may be a good alternative?
• The model in Figure 4 depicts an open hairpin conformation on the LD monolayer. This contrasts with simulations by Olarte et al. (2020, Dev Cell), but aligns with recent work (e.g., Dhiman et al., 2024 Nat Commun) showing open hairpins when UBXD8 moves onto the LD. The figure legend should acknowledge this prior work and discuss the potential role of seipin in modulating such conformational changes.
• While the authors highlight retrograde movement of LD proteins back to the ER as a novel finding, prior evidence exists (e.g., Dga1, Jacquier 2011 JCS; perilipins, Skinner et al., 2014 Adipocyte; Majchrzak et al., 2024 Cell Rep). This paper adds compelling demonstrating that seipin contact sites mediate such bidirectional transport, which is a major unexpected discovery.

Editing:
The axes labels and the panel titles in EDFig2a are too small.

Reviewer #2 (Remarks on code availability):

Code reviewing is beyond my core competence.

Reviewer #3 (Remarks to the Author):

The authors of this paper employ single molecule tracking to answer outstanding questions in protein trafficking. Specifically, the authors aim to provide support for a mechanism of localization of a subset of lipid droplet proteins. Despite decades of interest in the mechanisms that target and hold proteins to the phospholipid monolayer at the surface of lipid droplets, progress has been stalled by experimental limitations. The data presented by the authors contribute new insight into LD protein trafficking, using nanoscopy techniques that would likely be of high interest to the readers of Nature Cell Biology.

While the overall questions and approach are both original and significant, the following changes would improve the manuscript.

1. To increase accessibility to readers unfamiliar with the methods, the authors should be abundantly clear about what may seem like trivial or obvious points. For example, the authors may want to consider stating that differences in the anomalous diffusion exponents cannot be simply explained by the relatively smaller surface area of a single LD compared to the ER.

2. The authors conclude that LiveDrop accesses each LD through a single entry point. It’s not clear why Extended Data Fig 7c is cited as support for this conclusion. Moreover, the small number of examples shared in Extended Data Fig 7b are interesting but not enough to be conclusive. It’s particularly important to be careful about interpretation and include (somewhere) the caveot that LiveDrop is not likely representative of all LD proteins. The authors should consider stating clearly their opinions on how widely these results should be applied to localization of LD proteins, and address previously published models (such as in ref 7).

3. One model for protein targeting to LDs posits that LDs bud, but are not severed from the ER. Instead, a membrane connection that includes the seipin protein complex, may be retained. Protein may traffick to the LD by moving from the ER through this membrane bridge. The authors suggest that their data (Fig 3f) supports this model of LD protein trafficking. The four examples shown are certainly intriguing and could be a significant piece of mechanistic data. However, the authors fail to discuss the frequency at which the single protein tracks traverse a seipin enriched site. What about other recorded single molecule tracks of LiveDrop entering the LD surface? How can the reader be convinced that the apparent nearness of a patch of seipin density isn’t by chance? (It’s possible that Fig 3e or Extended Fig 10c are intended to provide additional support, but the individual traces are too small in these views to assess co-occurrence with seipin enriched patches.)

4. Given that there is evidence that LiveDrop can get to a subset of LDs in seipin KO cells, are the authors suggesting that transport through seipin anchored membrane bridges is one mode of LiveDrop localization or the mode of LiveDrop localization.

5. The authors assert that “seipin maintains a membrane bridge between ER and LDs that allows proteins to cross”. Evidence that LiveDrop moves across such a bridge is shown, but what’s the evidence that this bridge is maintained?

6. Some aspects of the model in Fig 4e are unclear. What is the significance of the salmon color? The figure legend includes the hypothesis that nanoscale domains are enriched is both triglycerides and phospholipids. The triglyceride piece is understandable, given the make-up of lipid droplets, but why would the LD nanodomain be enriched in phospholipids (as compared to the ER)? This model suggests a change in LiveDrop conformation. As that isn’t directly addressed by this paper, a reference (or further explanation) would be appropriate.

Editorial suggestions

1. The authors assert, “GPAT4’s speed of motion, reflected in the value of the jump distance and its apparent diffusion constant Dapp, was unaffected by the transition of the molecule from the ER bilayer to the LD monolayer”. Because it’s unclear if the small differences (Fig 1c and jump distance in Extended Fig 1e) are meaningful, this reviewer would prefer that the authors use similar or comparable, instead of “unaffected”.

2. Check that sample size is reported for all data sets. (For example, extended data Fig 1e, f; Fig 1g).

3. What LD cargo are data in Fig 1f? Legend states N=2155; how many were inside vs outside?

4. Check description of the calculation for density enrichment in the last sentence of Fig 1 legend. As stated, this does not make sense.

5. See paragraphs 3 and 4 in the section titled “The change in protein behavior on LDs is mediated by specific amino acid residues”. These are redundant paragraphs.

GUIDELINES FOR MANUSCRIPT SUBMISSION TO NATURE CELL BIOLOGY

READABILITY OF MANUSCRIPTS – Nature Cell Biology is read by cell biologists from diverse backgrounds, many of whom are not native English speakers. Authors should aim to communicate their findings clearly, explaining technical jargon that might be unfamiliar to non-specialists, and avoiding non-standard abbreviations. Titles and abstracts should concisely communicate the main findings of the study, and the background, rationale, results and conclusions should be clearly explained in the manuscript in a manner accessible to a broad cell biology audience. Nature Cell Biology uses British spelling.

MANUSCRIPT FORMAT – please follow the guidelines listed in our Guide to Authors regarding manuscript formats at Nature Cell Biology.

TITLE – should be no more than 100 characters including spaces, without punctuation and avoiding technical terms, abbreviations, and active verbs..

AUTHOR NAMES – should be given in full.

AUTHOR AFFILIATIONS – should be denoted with numerical superscripts (not symbols) preceding the names. Full addresses should be included, with US states in full and providing zip/post codes. The corresponding author is denoted by: "Correspondence should be addressed to [initials]."

ABSTRACT AND MAIN TEXT – please follow the guidelines that are specific to the format of your manuscript, as listed in our Guide to Authors (http://www.nature.com/ncb/pdf/ncb_gta.pdf) Briefly, Nature Cell Biology Articles, Resources and Technical Reports have 3500 words, including a 150 word abstract, and the main text is subdivided in Introduction, Results, and Discussion sections. Nature Cell Biology Letters have up to 2500 words, including a 180 word introductory paragraph (abstract), and the text is not subdivided in sections.

ACKNOWLEDGEMENTS – should be kept brief. Professional titles and affiliations are unnecessary. Grant numbers can be listed.

AUTHOR CONTRIBUTIONS – must be included after the Acknowledgements, detailing the contributions of each author to the paper (e.g. experimental work, project planning, data analysis etc.). Each author should be listed by his/her initials.

FINANCIAL AND NON-FINANCIAL COMPETING INTERESTS – the authors must include one of three declarations: (1) that they have no financial and non-financial competing interests; (2) that they have financial and non-financial competing interests; or (3) that they decline to respond, after the Author Contributions section. This statement will be published with the article, and in cases where financial and non-financial competing interests are declared, these will be itemized in a web supplement to the article. For further details please see https://www.nature.com/licenceforms/nrg/competing-interests.pdf.

REFERENCES – are limited to a total of 70 for Articles, Resources, Technical Reports; and 40 for Letters. This includes references in the main text and Methods combined. References must be numbered sequentially as they appear in the main text, tables and figure legends and Methods and must follow the precise style of Nature Cell Biology references. References only cited in the Methods should be numbered consecutively following the last reference cited in the main text. References only associated with Supplementary Information (e.g. in supplementary legends) do not count toward the total reference limit and do not need to be cited in numerical continuity with references in the main text. Only published papers can be cited, and

each publication cited should be included in the numbered reference list, which should include the manuscript titles. Footnotes are not permitted.

METHODS – Nature Cell Biology publishes methods online. The methods section should be provided as a separate Word document, which will be copyedited and appended to the manuscript PDF, and incorporated within the HTML format of the paper.

Methods should be written concisely, but should contain all elements necessary to allow interpretation and replication of the results. As a guideline, Methods sections typically do not exceed 3,000 words. The Methods should be divided into subsections listing reagents and techniques. When citing previous methods, accurate references should be provided and any alterations should be noted. Information must be provided about: antibody dilutions, company names, catalogue numbers and clone numbers for monoclonal antibodies; sequences of RNAi and cDNA probes/primers or company names and catalogue numbers if reagents are commercial; cell line names, sources and information on cell line identity and authentication. Animal studies and experiments involving human subjects must be reported in detail, identifying the committees approving the protocols. For studies involving human subjects/samples, a statement must be included confirming that informed consent was obtained. Statistical analyses and information on the reproducibility of experimental results should be provided in a section titled "Statistics and Reproducibility".

All Nature Cell Biology manuscripts submitted on or after March 21 2016 must include a Data availability statement as a separate section after Methods but before references, under the heading "Data Availability". . For Springer Nature policies on data availability see http://www.nature.com/authors/policies/availability.html; for more information on this particular policy see http://www.nature.com/authors/policies/data/data-availability-statements-data-citations.pdf. The Data availability statement should include:

• Accession codes for primary datasets (generated during the study under consideration and designated as "primary accessions") and secondary datasets (published datasets reanalysed during the study under consideration, designated as "referenced accessions"). For primary accessions data should be made public to coincide with publication of the manuscript. A list of data types for which submission to community-endorsed public repositories is mandated (including sequence, structure, microarray, deep sequencing data) can be found here http://www.nature.com/authors/policies/availability.html#data.

• Unique identifiers (accession codes, DOIs or other unique persistent identifier) and hyperlinks for datasets deposited in an approved repository, but for which data deposition is not mandated (see here for details http://www.nature.com/sdata/data-policies/repositories).

• At a minimum, please include a statement confirming that all relevant data are available from the authors, and/or are included with the manuscript (e.g. as source data or supplementary information), listing which data are included (e.g. by figure panels and data types) and mentioning any restrictions on availability.

• If a dataset has a Digital Object Identifier (DOI) as its unique identifier, we strongly encourage including this in the Reference list and citing the dataset in the Methods.

We recommend that you upload the step-by-step protocols used in this manuscript to protocols.io. More details can found at https://www.protocols.io/help/publish-articles.

DISPLAY ITEMS – main display items are limited to 6-8 main figures and/or main tables for Articles, Resources, Technical Reports; and 5 main figures and/or main tables for Letters. For Supplementary Information see below.

FIGURES – Colour figure publication costs $600 for the first, and $300 for each subsequent colour figure. All panels of a multi-panel figure must be logically connected and arranged as they would appear in the final version. Unnecessary figures and figure panels should be avoided (e.g. data presented in small tables could be stated briefly in the text instead).

All imaging data should be accompanied by scale bars, which should be defined in the legend.
Cropped images of gels/blots are acceptable, but need to be accompanied by size markers, and to retain visible background signal within the linear range (i.e. should not be saturated). The boundaries of panels with low background have to be demarked with black lines. Splicing of panels should only be considered if unavoidable, and must be clearly marked on the figure, and noted in the legend with a statement on whether the samples were obtained and processed simultaneously. Quantitative comparisons between samples on different gels/blots are discouraged; if this is unavoidable, it should only be performed for samples derived from the same experiment with gels/blots were processed in parallel, which needs to be stated in the legend.

Figures should be provided at approximately the size that they are to be printed at (single column is 86 mm, double column is 170 mm) and should not exceed an A4 page (8.5 x 11"). Reduction to the scale that will be used on the page is not necessary, but multi-panel figures should be sized so that the whole figure can be reduced by the same amount at the smallest size at which essential details in each panel are visible. In the interest of our colour-blind readers we ask that you avoid using red and green for contrast in figures. Replacing red with magenta and green with turquoise are two possible colour-safe alternatives. Lines with widths of less than 1 point should be avoided. Sans serif typefaces, such as Helvetica (preferred) or Arial should be used. All text that forms part of a figure should be rewritable and removable.

We accept files from the following graphics packages in either PC or Macintosh format:

- For line art, graphs, charts and schematics we prefer Adobe Illustrator (.AI), Encapsulated PostScript (.EPS) or Portable Document Format (.PDF). Files should be saved or exported as such directly from the application in which they were made, to allow us to restyle them according to our journal house style.

- We accept PowerPoint (.PPT) files if they are fully editable. However, please refrain from adding PowerPoint graphical effects to objects, as this results in them outputting poor quality raster art. Text used for PowerPoint figures should be Helvetica (preferred) or Arial.

- We do not recommend using Adobe Photoshop for designing figures, but we can accept Photoshop generated (.PSD or .TIFF) files only if each element included in the figure (text, labels, pictures, graphs, arrows and scale bars) are on separate layers. All text should be editable in 'type layers' and line-art such as graphs and other simple schematics should be preserved and embedded within 'vector smart objects' - not flattened raster/bitmap graphics.

- Some programs can generate Postscript by 'printing to file' (found in the Print dialogue). If using an application not listed above, save the file in PostScript format or email our Art Editor, Allen Beattie for advice (a.beattie@nature.com).

Regardless of format, all figures must be vector graphic compatible files, not supplied in a flattened raster/bitmap graphics format, but should be fully editable, allowing us to highlight/copy/paste all text and move individual parts of the figures (i.e. arrows, lines, x and y axes, graphs, tick marks, scale bars etc.). The only parts of the figure that should be in pixel raster/bitmap format are photographic images or 3D rendered graphics/complex technical illustrations.

All placed images (i.e. a photo incorporated into a figure) should be on a separate layer and independent from any superimposed scale bars or text. Individual photographic images must be a minimum of 300+ DPI (at actual size) or kept constant from the original picture acquisition and not decreased in resolution post image acquisition. All colour artwork should be RGB format.

EXTENDED DATA FIGURES - When re-submitting your manuscript, please ensure that any supplementary figures and tables that are crucial to the manuscript's conclusions are converted into Extended Data figures and tables to increase visibility of these data. Extended Data figures and tables are online-only (present in the online PDF and full-text HTML versions of the paper), peer-reviewed display items that provide essential background to the article but are not included in the main article due to space constraints. A maximum of ten Extended Data display items (figures and tables) is permitted.

FIGURE LEGENDS – must not exceed 350 words for each figure to allow fit on a single printed NCB page together with the figure. They must include a brief title for the whole figure, and short descriptions of each panel with definitions of the symbols used, but without detailing methodology.

TABLES – main tables should be provided as individual Word files, together with a brief title and legend. For supplementary tables see below.

SUPPLEMENTARY INFORMATION – Supplementary information is material directly relevant to the conclusion of a paper, but which cannot be included in the printed version in order to keep the manuscript concise and accessible to the general reader. Supplementary information is an integral part of a Nature Cell Biology publication and should be prepared and presented with as much care as the main display item, but it must not include non-essential data or text, which may be removed at the editor's discretion. All supplementary material is fully peer-reviewed and published online as part of the HTML version of the manuscript. Supplementary Figures and Supplementary Notes are appended at the end of the main PDF of the published manuscript.

Supplementary items should relate to a main text figure, wherever possible, and should be mentioned sequentially in the main manuscript, designated as Supplementary Figure, Table, Video, or Note, and numbered continuously (e.g. Supplementary Figure 1, Supplementary Figure 2, Supplementary Table 1, Supplementary Table 2 etc.).

Unprocessed scans of all key data generated through electrophoretic separation techniques need to be presented in a supplementary figure that should be labelled and numbered as the final supplementary figure, and should be mentioned in every relevant figure legend. This figure does not count towards the total number of figures and is the only figure that can be displayed over multiple pages, but should be provided as a single file, in PDF or TIFF format. Data in this figure can be displayed in a relatively informal style, but size markers and the figures panels corresponding to the presented data must be indicated.

The total number of Supplementary Figures (not including the "unprocessed scans" Supplementary Figure) should not exceed the number of main display items (figures and/or tables (see our Guide to Authors and March 2012 editorial http://www.nature.com/ncb/authors/submit/index.html#suppinfo; http://www.nature.com/ncb/journal/v14/n3/index.html#ed). No restrictions apply to Supplementary Tables or Videos, but we advise authors to be selective in including supplemental data.

Each Supplementary Figure should be provided as a single page and as an individual file in one of our accepted figure formats and should be presented according to our figure guidelines (see above). Supplementary Tables should be provided

as individual Excel files. Supplementary Videos should be provided as .avi or .mov files up to 50 MB in size. Supplementary Figures, Tables and Videos much be accompanied by a separate Word document including titles and legends.

GUIDELINES FOR EXPERIMENTAL AND STATISTICAL REPORTING

REPORTING REQUIREMENTS – We are trying to improve the quality of methods and statistics reporting in our papers. To that end, we are now asking authors to complete a reporting summary that collects information on experimental design and reagents. The Reporting Summary can be found here <a href="https://www.nature.com/documents/nr-reporting-summary.pdf">https://www.nature.com/documents/nr-reporting-summary.pdf</a>)If you would like to reference the guidance text as you complete the template, please access these flattened versions at <a href="http://www.nature.com/authors/policies/availability.html">http://www.nature.com/authors/policies/availability.html</a>.

STATISTICS – Wherever statistics have been derived the legend needs to provide the n number (i.e. the sample size used to derive statistics) as a precise value (not a range), and define what this value represents. Error bars need to be defined in the legends (e.g. SD, SEM) together with a measure of centre (e.g. mean, median). Box plots need to be defined in terms of minima, maxima, centre, and percentiles. Ranges are more appropriate than standard errors for small data sets. Wherever statistical significance has been derived, precise p values need to be provided and the statistical test used needs to be stated in the legend. Statistics such as error bars must not be derived from n<3. For sample sizes of n<5 please plot the individual data points rather than providing bar graphs. Deriving statistics from technical replicate samples, rather than biological replicates is strongly discouraged. Wherever statistical significance has been derived, precise p values need to be provided and the statistical test stated in the legend.

Information on how many times each experiment was repeated independently with similar results needs to be provided in the legends and/or Methods for all experiments, and in particular wherever representative experiments are shown.

We strongly recommend the presentation of source data for graphical and statistical analyses as a separate Supplementary Table, and request that source data for all independent repeats are provided when representative experiments of multiple independent repeats, or averages of two independent experiments are presented. This supplementary table should be in Excel format, with data for different figures provided as different sheets within a single Excel file. It should be labelled and numbered as one of the supplementary tables, titled “Statistics Source Data”, and mentioned in all relevant figure legends.

--------- Please don't hesitate to contact NCB@nature.com should you have queries about any of the above requirements ---------

Version 1:

Decision Letter:

Our ref: NCB-A59468A

12th March 2026

Dear Dr. Walther,

Thank you for submitting your revised manuscript "Membrane bridges and nanodomain partitioning govern targeting of membrane-embedded proteins to lipid droplets" (NCB-A59468A). It has now been seen by the original referees and their comments are below. Thank you for providing responses to Rev#1's remaining points, which Rev#1 commented on below. The reviewers find that the paper has improved in revision, and therefore we'll be happy in principle to publish it in Nature Cell Biology, pending minor revisions to satisfy the referees' final requests and to comply with our editorial and formatting guidelines. Please do follow Rev#1's guidance for the final changes needed (below).

The current version of your manuscript is in a PDF format; could you please email us a copy of the file in an editable format (Microsoft Word) as we can not proceed with PDFs at this stage? Thank you in advance.

We are now performing detailed checks on your paper and will send you a checklist detailing our editorial and formatting requirements in 1-2 weeks from the time of reception of the Word file. Please do not upload the final materials and make any revisions until you receive this additional information from us.

Thank you again for your interest in Nature Cell Biology. Please do not hesitate to contact me if you have any questions.

Sincerely,

Melina Casadio, PhD
Senior Editor, Nature Cell Biology
ORCID ID: https://orcid.org/0000-0003-2389-2243

Reviewer #1 (Remarks to the Author):

The authors have adequately addressed my raised issues and are commended for their thorough revisions.

Remaining minor comments (related to Reviewer 1 point-by-point address):

2. I strongly recommend showing colocalization analysis of the results in Extended Data Fig. 6D to support the colocalization of the freely diffusive molecules with the ER marker. How does this colocalization change for the LD-localized trajectories?

3. Extended Data Fig 2c, 2d seem to relate to HSD17B13. Do the authors relate to other panels in the figure (e, f, or g), or is there some other mistake in labelling?

4. Extended Fig. 1i – please calculate and mention a correlation measure (e.g., Pearson's) to show that Dapp and alpha have no meaningful correlation.

7. The authors seem to refer to the new Extended Fig. 1g; right?!

9. I think that the VVAC results for the LD population are interesting and worth showing to support the conclusions of the manuscript and to readers interested in molecular diffusion. Indeed, these results show strong evidence of confinement as a prime mechanism (i.e., long-term / "long-range constraints on motion") since VVAC is one of the most revealing statistical tests for distinguishing (sub)diffusion mechanisms [e.g., Golan et al, Nature Communications volume 8, Article number: 15851 (2017)]

ADDITIONAL COMMENTS ON AUTHORS' RESPONSES:

I strongly recommend mentioning the relevant measures and results in points 2, 4, and 9 in the text. Specifically, point 9 deserves mentioning in the main text.
With these minor changes, the manuscript should be ready for acceptance.

Reviewer #2 (Remarks to the Author):

The authors have addressed most points raised by this reviewer and the work should be published without further changes.

Reviewer #2 (Remarks on code availability):

This falls beyond my primary expertise.

Reviewer #3 (Remarks to the Author):

The authors have adequately addressed the concerns raised in my review, including the request for additional clarity regarding sample sizes and data interpretation. The revised manuscript presents data in support of an intriguing model of nanodomain partitioning of lipid droplet proteins and documents single molecule movement between the ER and lipid droplet. In my opinion, this model and supporting data would be of significant interest to readers of this journal.

Version 2:

Decision Letter:

Dear Dr Walther,

I am pleased to inform you that your manuscript, "Membrane bridges and nanodomain partitioning govern membrane protein targeting to lipid droplets", has now been accepted for publication in Nature Cell Biology.

Thank you for sending us the final manuscript files to be processed for print and online production, and for returning the manuscript checklists and other forms. Your manuscript will now be passed to our production team who will be in contact with you if there are any questions with the production quality of supplied figures and text.

Over the next few weeks, your paper will be copyedited to ensure that it conforms to Nature Cell Biology style. Once your paper is typeset, you will receive an email with a link to choose the appropriate publishing options for your paper and our Author Services team will be in touch regarding any additional information that may be required.

After the grant of rights is completed, you will receive a link to your electronic proof via email with a request to make any corrections within 48 hours. If, when you receive your proof, you cannot meet this deadline, please inform us at rjsproduction@springernature.com immediately.

You will not receive your proofs until the publishing agreement has been received through our system.

Due to the importance of these deadlines, we ask that you please let us know now whether you will be difficult to contact over the next month. If this is the case, we ask you provide us with the contact information (email, phone and fax) of someone who will be able to check the proofs on your behalf, and who will be available to address any last-minute problems.

If you have any questions about our publishing options, costs, Open Access requirements, or our legal forms, please contact ASJournals@springernature.com

Once your paper has been scheduled for online publication, the Nature press office will be in touch to confirm the details. An online order form for reprints of your paper is available at <a href="https://www.nature.com/reprints/author-reprints.html">https://www.nature.com/reprints/author-reprints.html</a>. All co-authors, authors' institutions and authors' funding agencies can order reprints using the form appropriate to their geographical region.

Publication is conditional on the manuscript not being published elsewhere and on there being no announcement of this work to any media outlet until the online publication date in Nature Cell Biology.

You may wish to make your media relations office aware of your accepted publication, in case they consider it appropriate to organize some internal or external publicity. Once your paper has been scheduled you will receive an email confirming the publication details. This is normally 3-4 working days in advance of publication. If you need additional notice of the date and time of publication, please let the production team know when you receive the proof of your article to ensure there is sufficient time to coordinate. Further information on our embargo policies can be found here: https://www.nature.com/authors/policies/embargo.html

Please note that *Nature Cell Biology* is a Transformative Journal (TJ). Authors may publish their research with us through the traditional subscription access route or make their paper immediately open access through payment of an article-processing charge (APC). Authors will not be required to make a final decision about access to their article until it has been accepted. <a href="https://www.springernature.com/gp/open-research/transformative-journals"> Find out more about Transformative Journals</a>

**Authors may need to take specific actions to achieve compliance with funder and institutional open access mandates.** If your research is supported by a funder that requires immediate open access (e.g. according to <a href="https://www.springernature.com/gp/open-science/plan-s-compliance"> Plan S principles</a> or the <a href="https://www.springernature.com/gp/open-science/us-federal-agency-compliance"> NIH public access policy</a>) then you should select the gold OA route, and we will direct you to the compliant route where possible. Because authors warrant under our subscription licensing terms that they haven't committed to licensing any version of their article under a licence inconsistent with the terms of our agreement – including the applicable embargo period – publication under the subscription model isn't suitable for authors whose funders require no embargo.

To assist our authors in disseminating their research to the broader community, our SharedIt initiative provides you with a unique shareable link that will allow anyone (with or without a subscription) to read the published article. Recipients of the link with a subscription will also be able to download and print the PDF.

If your paper includes color figures, please be aware that in order to help cover some of the additional cost of four-color reproduction, Nature Portfolio charges our authors a fee for the printing of their color figures. Please contact our offices for exact pricing and details.

As soon as your article is published, you will receive an automated email with your shareable link.

If you have not already done so, we strongly recommend that you upload the step-by-step protocols used in this manuscript to protocols.io (https://protocols.io), an open online resource that allows researchers to share their detailed experimental know-how. All uploaded protocols are made freely available and are assigned DOIs for ease of citation. Protocols and Nature Portfolio journal papers in which they are used can be linked to one another, and this link is clearly and prominently visible in the online versions of both. Authors who performed the specific experiments can act as primary authors for the Protocol as they will be best placed to share the methodology details, but the Corresponding Author of the present research paper should be included as one of the authors. By uploading your Protocols onto protocols.io, you are enabling researchers to more readily reproduce or adapt the methodology you use, as well as increasing the visibility of your protocols and papers. You can also establish a dedicated workspace to collect your lab Protocols. Further information can be found at https://www.protocols.io/help/publish-articles.

You can use a single sign-on for all your accounts, view the status of all your manuscript submissions and reviews, access usage statistics for your published articles and download a record of your refereeing activity for the Nature Portfolio.

Nature Cell Biology encourages authors presenting evidence for cell, biological, molecular, and genetic interactions to

consider communicating these findings using Biofactoid (https://biofactoid.org/). This tool helps users share a searchable representation of interactions (e.g. binding, gene expression, post-translational modification) between genes, gene products, or chemicals. Information added to Biofactoid, with author attribution, is shared on social media and public databases, such as Pathway Commons, where it can be discovered and analyzed in the context of a large and growing corpus of knowledge.

Please feel free to contact us if you have any questions.

With kind regards,

Melina Casadio, PhD
Senior Editor, Nature Cell Biology
ORCID ID: https://orcid.org/0000-0003-2389-2243

Click here if you would like to recommend Nature Cell Biology to your librarian
http://www.nature.com/subscriptions/recommend.html#forms

** Visit the Springer Nature Editorial and Publishing website at <a href="http://editorial-jobs.springernature.com?utm_source=ejP_NCB_email&utm_medium=ejP_NCB_email&utm_campaign=ejp_NCB">www.springernature.com/editorial-and-publishing-jobs</a> for more information about our career opportunities. If you have any questions please click <a href="mailto:editorial.publishing.jobs@springernature.com">here</a>.**

**Open Access** This Peer Review File is licensed under a Creative Commons Attribution 4.0 International License, which permits use, sharing, adaptation, distribution and reproduction in any medium or format, as long as you give appropriate credit to the original author(s) and the source, provide a link to the Creative Commons license, and indicate if changes were made.
In cases where reviewers are anonymous, credit should be given to 'Anonymous Referee' and the source.
The images or other third party material in this Peer Review File are included in the article’s Creative Commons license, unless indicated otherwise in a credit line to the material. If material is not included in the article’s Creative Commons license and your intended use is not permitted by statutory regulation or exceeds the permitted use, you will need to obtain permission directly from the copyright holder.
To view a copy of this license, visit https://creativecommons.org/licenses/by/4.0/

**RESPONSE TO REVIEWERS**

**Reviewer #1**

Mizrak et al study the dynamics of membrane proteins that shuttle between the ER and lipid droplets (LDs). They employ cutting-edge microscopy (primarily MINFLUX) to follow single proteins in the cells. They recognize confinement in nanodomains on LDs and bridge crossing between the ER and LDs as mechanisms of prime interest. Despite these findings and advanced imaging technique, I have major reservations regarding how the data is analyzed and interpreted:

We thank the reviewer evaluating our manuscript and for providing constructive feedback. We believe this substantially strengthened our data analyses and enhanced the statistical grounding of our conclusions. While the overall biological conclusions remain unchanged, they are now more robustly supported by quantitative analyses, and the manuscript is clearer and stronger as a result. We address each point raised by the reviewer in detail below.

1. Fig. 1 – The authors should first show the entire cell.

We agree and have added the confocal images of the entire cells that are stained for the lipid dye BODIPY 493/503 (Fig 1a,b).

2. Highlights of the ER are expected, while shown only for LDs

We agree with the reviewer that highlighting ER regions would be informative. Although we did not perform MINFLUX imaging with a dedicated ER marker, our HILO-based single-molecule tracking experiments provide strong support for ER assignment. Specifically, trajectories classified as outside the LD mask consistently colocalize with an ER marker (Extended Data Fig. 6d), and their diffusion properties exhibit free motion with higher α values than of LD-localized trajectories. Together, the spatial colocalization and diffusion behavior give us confidence that tracks outside the BODIPY-defined LD regions correspond to ER-localized populations, allowing reliable classification of ER versus LD trajectories in our analysis.

3. The distributions of D and alpha are very broad for both proteins under study. Why? Can the authors segment the trajectories to distinguish different mobility characteristics within single trajectories?

We thank the reviewer for this important point. Gaussian mixture modeling of the *Dapp* distributions reveals two mobility states: a slow-diffusing state and a faster-diffusing state (Extended Fig 2a). We also observe that the slow mobility is selectively enriched within nanodomains, consistent with our KDE analysis showing that nanodomain clustering restricts mobility. A new supplementary panel (Extended Data Fig 2c, 2d) now compares these mobility-state distributions for GPAT4 and HSD17B13 and relates them to nanodomain localization.

4. Is there a correlation between D and alpha values? Show a 2D map of these parameters for individual trajectories.

When alpha values are computed over 5 ms sliding windows, the short timescale results in alpha ~1 for most segments, largely independent of the corresponding *Dapp*. We also do not observe a meaningful correlation between trajectory-averaged sliding-window *Dapp* and track-level alpha values. Following the reviewer's suggestion, we have added a 2D map of trajectory-averaged *Dapp* vs alpha for both LD-associated proteins to the supplementary materials (Extended Fig. 1i).

5. A main concern relates to the definition of the nanodomains. Could it be that there is excluded volume on the LDs? How does its 3D shape affect the appearance of the trajectories? Can the authors track molecules in 3D or provide 3D cell images, as they employ spinning disk confocal imaging in the study?

We thank the reviewer for raising this important point. We consider it unlikely that nanodomains represent excluded-volume regions, as these sites are characterized by increased local trapping and reduced mobility rather than by avoidance. In our data, proteins repeatedly enter these regions and exhibit prolonged residence times with slowed diffusion, which is not consistent with exclusion and instead supports local retention. Whether nanodomain regions also correspond to higher local protein concentration or molecular crowding is an intriguing question, but we do not know how to address this currently.

Unfortunately, our current setup does not allow direct three-dimensional single-molecule tracking (which remains an important technology goal for the future). However, the differential behavior of WT and 3W mutants with similar membrane geometry argue against 3D shape as the origin of nanodomain formation.

These considerations led us to propose a model with nanoscale packing defects and local presentation of neutral lipids at the aqueous interface modulating protein motion, with conserved tryptophan residues fine-tuning interactions within these regions and thereby driving global accumulation. We have edited the discussion to more clearly explain limitations of our study and conclusions.

6. Panel f – what are the associated errors in C and D? Are the differences in D values significant?

We thank the reviewer for this comment and quantified differences in *Dapp* and alpha values using nonparametric bootstrap resampling to estimate the uncertainty in the median and assessed statistical significance with Mann–Whitney and Kolmogorov–Smirnov tests. The resulting medians, 95% confidence intervals, and p-values are reported in the legends of Fig. 1c and Extended Data Fig. 1d.

Our analyses show statistically detectable differences in both *Dapp* and alpha values between ER and LD-localized molecules. Notably, although the median *Dapp* values for GPAT4 at LD and ER differ by only ~0.0037 $\mu m^2/s$ (~4.7%), this small shift appears statistically significant (Mann Whitney $p=4\times10^{-27}$) due to the large number of analyzed steps ($N_{steps}$= 551,673).

7. Extended Fig. 1, panel f – How do the graphs look like using log-log representation?

The ER and LD trajectories exhibit similar slopes at short lag times (up to ~50 ms), indicating comparable short-range diffusion. At longer lag times, however, the slopes begin to diverge, reflecting differences in anomalous diffusion behavior. We have added the log–log MSD presentation as Extended Fig. 1f.

8. Are the different random walks ergodic under the various conditions? The authors should conduct both time and ensemble averaging of MSDs and compare them.

We compared the ensemble-averaged MSD with trajectory-wise time-averaged MSD across all lag times both for the full dataset and separately for ER- and LD-localized trajectories for each LD protein. In all cases, ensemble-averaged and time-averaged MSD overlap across lag times, indicating that the dynamics are effectively ergodic under the measured conditions. Thus, the reduced alpha values observed on LDs reflect a change in the local diffusion regime (e.g., increased confinement or transient mobility change upon nanodomain-association) rather than a non-ergodic trapping process. We include this comparison in Extended Data Fig. 1h.

9. The authors are advised to employ velocity-velocity autocorrelation to further inspect differences in mobility mechanisms.

We thank the reviewer for this suggestion. We performed velocity-velocity autocorrelation for GPAT4 and HSD17B13 to assess the mobility mechanisms at the ER and LDs for all the tracks we have recorded (shown only for the reviewer, below). At short timescales (e.g., window = 5 ms), velocity autocorrelation rapidly decays toward

zero for both ER- and LD-localized molecules, consistent with largely random, diffusion-like motion and minimal confinement. As the observation window increases, LD-localized molecules exhibit lower VVAC function values, earlier zero-crossing, and stronger negative correlations than ER-localized molecules, reflecting increasing velocity reversals and long-range constraints on motion. At time scale increases (window = 50ms), this divergence is most pronounced, with sustained anti-persistent motion on LDs accompanied by a progressive decrease in α, indicating cumulative confinement despite similar instantaneous diffusion speeds.

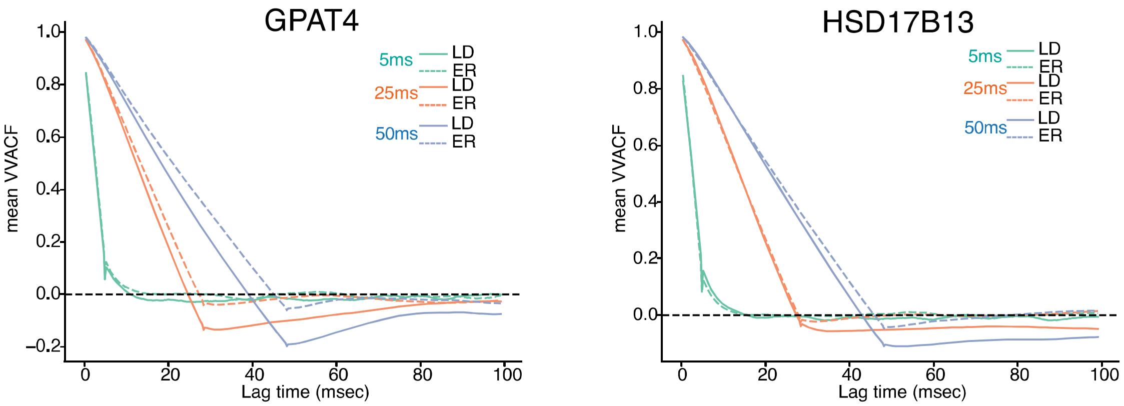


10. Fig. 2 – It is hard to evaluate and thus unclear whether the protein mutations have a significant effect, as no errors are shown for panels b,e,h,k. Overall, these effects seem quite modest.

We agree with the reviewer and have revised our analysis to include quantitation of the localization fractions per cell and now report mean values ± SEM across biological replicates. Statistical comparisons between WT and mutant conditions were performed using appropriate test (Extended Data Fig 3h - legend). These analyses confirm that the progressive increase in ER-localized trajectories and corresponding decrease in LD-localized trajectories observed for the 3W and 3W+KRR mutants are modest in magnitude but statistically significant.

We also clarify in the text that these effects reflect shifts in partitioning probability rather than binary localization outcomes, consistent with a model in which the mutations alter nanodomain engagement and retention on LDs rather than abolishing LD access entirely.

11. How were graphs in panels c,f,I,l normalized?

The y-axis represents the probability density, normalized for the total area under each histogram equaling 1. Normalization was performed independently for each group to allow direct comparison distribution shapes.

12. Fig. 3 – A distinct and independent marker for ER is expected, not just for LDs.

We agree and have added a supplementary panel in Extended Fig. 6d showing the ER marker in green together with single-molecule tracks of *LiveDrop*, confirming that the non-LD trajectories reside on the ER.

13. What are the potential issues of cell treatment with oleic acids?

This is another good point. Oleic acid treatment increases cellular triglyceride stores and can alter membrane composition by stimulating phospholipid synthesis, which in principle could influence membrane fluidity and protein mobility. However, we observe comparable diffusion of *LiveDrop* in both conditions, indicating that oleic acid treatment does not measurably affect the mobility parameters analyzed in this study.

14. No statistics are shown

We agree with the reviewer that statistical analysis is important to assess the significance of our results. To this effect, we segmented LD-like structures from *Livedrop* density maps and seipin-enriched ER regions from seipin density maps, then assigned single-molecule diffusion breakpoints to in-seipin, near-seipin, or seipin-not-detected regions. After normalizing breakpoint counts to the number of LD-like objects per sample, we found that ER–LD–travelling molecules show a clear enrichment of state-change breakpoints at seipin sites, indicating that seipin marks preferred ER–LD transition zones. We included the tables with standard deviations and statistical tests in the Extended Data Fig 10.

15. Fig. 4 – No statistics are shown.

We have now added statistical analyses for reverse (LD-to-ER) trafficking using both confocal imaging–based measurements (Extended Data Fig 12) and single-molecule tracking (Extended Data Fig 10). We observe comparable frequencies and spatial distributions of inward and outward transitions, including similar enrichment of diffusion breakpoints relative to seipin-enriched regions, indicating that the reverse trafficking behavior follows the same spatial principles as forward ER-to-LD trafficking.

16. The scheme in Panel e is very speculative. No evidence is provided for the conformational and orientation changes.

We agree that our data do not directly address conformational or orientational changes of the *LiveDrop* hairpin. We have revised Fig. 4e, its legend and the discussion to better reflect our conclusions and to clearly explain that Seipin allows bidirectional trafficking and nanoscale membrane environments influence protein mobility and confinement.

17. ED Fig. 8a shows seipin clusters. Could trapping seipin in such protein clusters be the main mechanism of confinement? How can this mechanism be excluded?

We consider it unlikely that confinement arises primarily from *LiveDrop* being trapped within seipin protein because there are multiple spatially distinct nanodomains on individual LDs but only one seipin cluster. We have edited the manuscript to better reflect these points.

**Reviewer #2**

The manuscript by Mizrak et al. represents a technical tour de force that clarifies several fundamental aspects of protein targeting to lipid droplets (LDs) in mammalian cells. The central advance is the direct, quantitative characterization of ER-to-LD partitioning in live cells using a model cargo, "live drop." Live drop consists of the hairpin membrane anchor of GPAT4, a lipid enzyme that dynamically partitions between the ER and LDs. Coupled with automated, machine learning–based trace analysis, this work provides, to my knowledge, the first unbiased quantitative investigation of protein targeting to LDs in live cells.
Key findings include the demonstration that the membrane anchors of GPAT4, HSD17B13, and live drop govern ER–LD partitioning and form previously unrecognized nanodomains on the LD surface that mediate LD trapping. The data further establish that these membrane proteins access LDs from the ER through seipin-marked contact sites, which appear to enable bidirectional transport.

The data and figures are of exceptional quality, and the conclusions are clearly presented. This study has the potential to become a "classic" in the LD field and beyond. I strongly support publication in Nature Cell Biology, contingent upon revision of the following points:

We thank the reviewer for her/his/their thoughtful comments and for recognizing this work. A detailed answer to the feedback is below.

Experimental queries:

1. The study introduces full-length GPAT4, HSD17B13, and PNPLA3 as cargoes of interest, but does not test whether seipin loss alters their partitioning. After the live drop analysis, it would be important to characterize at least one of these full-length cargoes in seipin knockout cells.

We agree and thank the reviewer for this excellent point. Full length cargoes are indeed also affected in cells lacking seipin. We have included the data in Extended Data Fig 11 to demonstrate the partitioning defects for HSD17B13 in the absence of seipin. We made extensive efforts to establish RUSH assays for full length proteins. However, it appears hard to retain them in functional states and they rather form what appears to be aggregates close to LDs (shown for the reviewer, below), preventing further analysis with this assay.

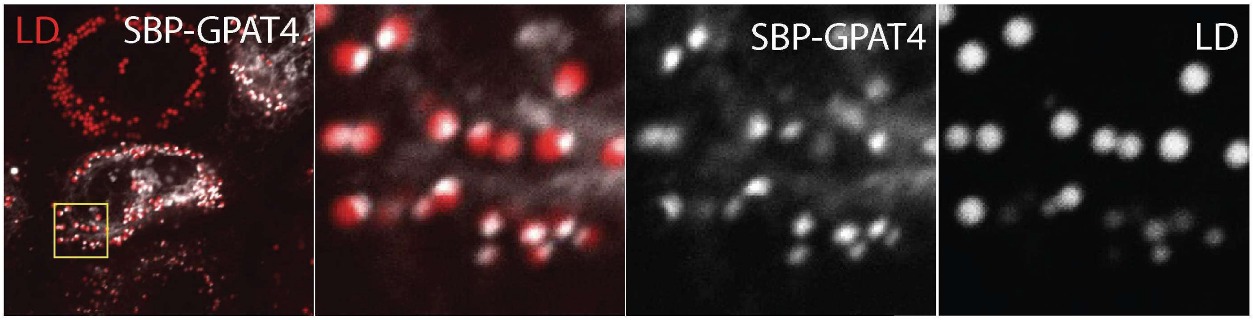


2. Alternatively, it would be informative to assess whether KRR or WW mutations exhibit defects in nanodomain trapping similar to live drop. Would such results help explain the LD phenotypes observed in seipin loss-of-function models?

We agree, have performed this analysis and it is included in the revised manuscript. KRR and WW substitutions alter LD localization by disrupting how the hairpins engage LD nanodomains. Loss of tryptophans does not reduce nanodomain encounters per se but without forming persistent clusters seen for the wildtype protein (Fig. 2).

In seipin-deficient cells, only a subset of LDs receive *LiveDrop,* most consistent with impaired physical continuity between the ER and LD membranes.

3. The conformational changes of live drop presented in Figure 4 require clarification. The proposed model contradicts Olarte et al. (2020, Dev Cell), which described the hairpin as remaining "closed." Could the authors provide additional simulations or rationale supporting their interpretation?

We agree that our data do not directly address conformational or orientational changes of the *LiveDrop* hairpin. We have revised Fig. 4e, its legend and the discussion to better reflect our conclusions and to clearly illustrate that seipin allows bidirectional trafficking and nanoscale membrane environments influence protein mobility and confinement.

Conceptual comments

4. The Walther/Farese group previously introduced GPAT4 as a late ER-to-LD cargo that targets LDs through seipin-independent bridges (e.g., Song et al., 2022 NCB; Olarte et al., 2022 Trends Biochem Sci; Wilfling et al., 2013 Dev Cell). This manuscript now suggests GPAT4 enters LDs via seipin-bridges. The mixing between

“bridges” and “seipin contact sites” may confuse readers, as these have previously been explicitly distinguished. The authors should clarify whether this reflects cell-type specificity (e.g., flies versus mammals) or whether live drop behaves differently because it contains only a single LD-targeting domain.

We agree and have clarified this point in the revised manuscript. Consistent with the previous work, *LiveDrop* access LDs early during their formation, and through *seipin* contacts. Analysis of later targeting events of full-length cargoes is complicated by experimental difficulties of RUSH assays for these cargoes and will require further analysis.

5. The title “Organelle bridges…” is somewhat misleading, as this study focuses exclusively on ER–LD contact sites. A more precise title, such as “ER contact sites and nanodomain partitioning…”, may be a good alternative?

We agree, are grateful for the suggestion and have in response revised the title to ‘*Membrane bridges and nanodomain partitioning govern targeting of membrane-embedded proteins to lipid droplets*,’ to more accurately reflect our focus on ER–LD contacts.

6. The model in Figure 4 depicts an open hairpin conformation on the LD monolayer. This contrasts with simulations by Olarte et al. (2020, Dev Cell), but aligns with recent work (e.g., Dhiman et al., 2024 Nat Commun) showing open hairpins when UBXD8 moves onto the LD. The figure legend should acknowledge this prior work and discuss the potential role of seipin in modulating such conformational changes.

We agree and have made sure to include the reference in the revised figure which more clearly outlines the key finding of nanodomains with altered protein mobility on LDs.

7. While the authors highlight retrograde movement of LD proteins back to the ER as a novel finding, prior evidence exists (e.g., Dga1, Jacquier 2011 JCS; perilipins, Skinner et al., 2014 Adipocyte; Majchrzak et al., 2024 Cell Rep). This paper adds compelling demonstrating that seipin contact sites mediate such bidirectional transport, which is a major unexpected discovery.

We thank the reviewer for pointing this out and have modified the paper in response. Previously, most data tested re-localization of proteins to the ER when conditions change (such as TG depletion). We now add more direct evidence that LD-resident proteins undergo retrograde exchange under *steady-state* conditions.

Editing:
The axes labels and the panel titles in ED Fig2a are too small.

Thank you for bringing this to our attention. We have fixed the axes labels.

Reviewer #2 (Remarks on code availability):

Code reviewing is beyond my core competence.

**Reviewer #3**

The authors of this paper employ single molecule tracking to answer outstanding questions in protein trafficking. Specifically, the authors aim to provide support for a mechanism of localization of a subset of lipid droplet proteins. Despite decades of interest in the mechanisms that target and hold proteins to the phospholipid monolayer at the

surface of lipid droplets, progress has been stalled by experimental limitations. The data presented by the authors contribute new insight into LD protein trafficking, using nanoscopy techniques that would likely be of high interest to the readers of Nature Cell Biology.

While the overall questions and approach are both original and significant, the following changes would improve the manuscript.

We thank the reviewer for their support and appreciate the constructive feedback. In response, we have revised the manuscript to address all these valuable suggestions.

1. To increase accessibility to readers unfamiliar with the methods, the authors should be abundantly clear about what may seem like trivial or obvious points. For example, the authors may want to consider stating that differences in the anomalous diffusion exponents cannot be simply explained by the relatively smaller surface area of a single LD compared to the ER.

We thank the reviewer for this helpful suggestion. We have revised the text to improve accessibility by explicitly motivating the anomalous diffusion analysis and clarifying that the observed differences in anomalous diffusion exponents are not explained solely by geometry, but instead reflect changes in the local diffusion regime.

2. The authors conclude that *LiveDrop* accesses each LD through a single entry point. It's not clear why Extended Data Fig 7c is cited as support for this conclusion. Moreover, the small number of examples shared in Extended Data Fig 7b are interesting but not enough to be conclusive. It's particularly important to be careful about interpretation and include (somewhere) the caveat that *LiveDrop* is not likely representative of all LD proteins. The authors should consider stating clearly their opinions on how widely these results should be applied to localization of LD proteins, and address previously published models (such as in ref 7).

We thank the reviewer and agree on all points. First, we apologize for mis-citing Extended Figure 7C. The data showing a single LD entry point is shown in Extended Figure 6 (we also revised the manuscript to clarify this). Second, we appreciate cautioning us regarding overinterpretation of the data. ER-to-LD transition is a rare event and we confidently captured a few hundred (in thousands of tracks). This of course might limit the possibility to observe alternative targeting modes, and we now discuss this limitation of our approach. Third, we more clearly address the differences of *LiveDrop* and full-length cargo, which unfortunately has proved much harder to analyze (e.g., RUSH leads to protein aggregation in the ER or partial targeting).

3. One model for protein targeting to LDs posits that LDs bud, but are not severed from the ER. Instead, a membrane connection that includes the seipin protein complex, may be retained. Protein may traffic to the LD by moving from the ER through this membrane bridge. The authors suggest that their data (Fig 3f) supports this model of LD protein trafficking. The four examples shown are certainly intriguing and could be a significant piece of mechanistic data. However, the authors fail to discuss the frequency at which the single protein tracks traverse a seipin enriched site. What about other recorded single molecule tracks of *LiveDrop* entering the LD surface? How can the reader be convinced that the apparent nearness of a patch of seipin density isn't by chance? (It's possible that Fig 3e or Extended Fig 10c are intended to provide additional support, but the individual traces are too small in these views to assess co-occurrence with seipin enriched patches.)

We thank the reviewer, agree and have struggled with this point. We now discuss the frequency of events in the revised manuscript to outline limitations of the experiment (e.g., due to relatively small number of very rare events observed). To address whether the change in diffusion pattern at seipin is by change, we calculated the likelihood of that diffusion change happening at a random point and asked whether the null hypothesis (a random change close to seipin) can be rejected- which it was. We observed significant enrichment of diffusion breakpoints (indicative of LD entry/exit) for tracks that are travelling between the ER and LDs at seipin containing regions compared to regions where seipin was not detected. The table and statistical analysis is now included in Extended Data Fig 10.

4. Given that there is evidence that *LiveDrop* can get to a subset of LDs in seipin KO cells, are the authors suggesting that transport through seipin anchored membrane bridges is one mode of *LiveDrop* localization or the mode of *LiveDrop* localization.

We thank the reviewer for this comment and have clarified in the revised manuscript that our data support that transport through seipin is at least one mode of transport, that we do not find evidence for other modes, and that if this occurred somewhat more rarely we might have missed it.

5. The authors assert that "seipin maintains a membrane bridge between ER and LDs that allows proteins to cross". Evidence that *LiveDrop* moves across such a bridge is shown, but what's the evidence that this bridge is maintained?

We have clarified this in the paper that this is based on the following observations as the most parsimonious interpretation: 1) we observe *LiveDrop* migrating to LDs through seipin in MINFLUX 2) we observe *LiveDrop* can access LDs when released from RUSH hooks with identical kinetics at any time point of LD formation. We clarify that a persistent bridge is one interpretation, but that rapidly forming and dissolving bridges would be an alternative we cannot currently rule out).

6. Some aspects of the model in Fig 4e are unclear. What is the significance of the salmon color? The figure legend includes the hypothesis that nanoscale domains are enriched is both triglycerides and phospholipids. The triglyceride piece is understandable, given the make-up of lipid droplets, but why would the LD nanodomain be enriched in phospholipids (as compared to the ER)? This model suggests a change in *LiveDrop* conformation. As that isn't directly addressed by this paper, a reference (or further explanation) would be appropriate.

We agree that our data do not directly address conformational or orientational changes of the *LiveDrop* hairpin. We have revised Fig. 4e and its legend to better reflect our conclusions and to clearly illustrate that Seipin allows bidirectional trafficking and nanoscale membrane environments influence protein mobility and confinement.

**Editorial suggestions**

1. The authors assert, "GPAT4's speed of motion, reflected in the value of the jump distance and its apparent diffusion constant *Dapp*, was unaffected by the transition of the molecule from the ER bilayer to the LD monolayer". Because it's unclear if the small differences (Fig 1c and jump distance in Extended Fig 1e) are meaningful, this reviewer would prefer that the authors use similar or comparable, instead of "unaffected".

We thank you for this valid point. The differences in mobility between ER- and LD-localized statistically significant, despite being very small. We have revised the text to state that the $D_{app}$ values are similar between ER- and LD-localized proteins and provide the quantitative data.

2. Check that sample size is reported for all data sets. (For example, extended data Fig 1e, f; Fig 1g).

Sample sizes for datasets used in each analysis are now included in the corresponding figure legends.

3. What LD cargo are data in Fig 1f? Legend states N=2155; how many were inside vs outside?

Thank you for bringing this to our attention, and we apologize for the ambiguity. We have corrected the number of tracks analyzed in Fig. 1f and clarified which LD cargo is plotted in the figure.

4. Check description of the calculation for density enrichment in the last sentence of Fig 1 legend. As stated, this does not make sense.

We have revised the description of density enrichment and now refer to this metric as a *clustering index* to more accurately reflect what is being quantified.

5. See paragraphs 3 and 4 in the section titled “The change in protein behavior on LDs is mediated by specific amino acid residues”. These are redundant paragraphs.

We have removed the redundant paragraph and clarified the corresponding section.

**Points raised by the reviewer –** our response:

**2. I strongly recommend showing colocalization analysis of the results in Extended Data Fig. 6D to support the colocalization of the freely diffusive molecules with the ER marker. How does this colocalization change for the LD-localized trajectories?**

We thank the reviewer for this suggestion. Colocalization analysis with ER and LD markers shows that 96 ± 1.5% of freely diffusive trajectories (N = 975) localize to ER regions, with only 4 ± 1.5% observed on LDs. In contrast, 70 ± 1.2% of confined trajectories (N = 502) localize to LDs, whereas 29 ± 1.2% colocalize with the ER. Importantly, when restricting the analysis to high-confidence trajectories, we do not observe tracks that fall outside either the ER or LD masks, indicating that the detected molecules are robustly associated with these membrane compartments. These results quantitatively support the visual overlay shown in Extended Data Fig. 6d and are consistent with the interpretation that freely diffusive molecules predominantly track along ER membranes, while confined trajectories are enriched on LDs.

We would be happy to include this colocalization analysis and the associated statistical summary in the revised manuscript (either in the main text or Extended Data), if the editor and reviewer feel it would be helpful for readers.

**3. Extended Data Fig 2c, 2d seem to relate to HSD17B13. Do the authors relate to other panels in the figure (e, f, or g), or is there some other mistake in labelling?**

We have incorporated the GPAT4 related data into the main manuscript (Fig. 1f,g), while the corresponding HSD17B13 data were presented in Extended Data Fig. 2c,d. We chose to highlight GPAT4 in the main figure for clarity and simplicity, as the results for both proteins are highly consistent. However, if the editor or reviewer feels that it would be helpful, we would be happy to move the HSD17B13 data into the main figure as well.

**4. Extended Fig. 1i – please calculate and mention a correlation measure (e.g., Pearson's) to show that Dapp and alpha have no meaningful correlation.**

We thank the reviewer for this suggestion. We performed a Pearson correlation analysis between the apparent diffusion coefficient ($D_{app}$) and the anomalous exponent ($\alpha$). This analysis revealed no significant relationship between the two variables (GPAT4: $r = -0.09$, $P = 0.15$; HSD17B13: $r = -0.03$, $P = 0.53$). These results indicate that the apparent diffusion coefficient and anomalous exponent vary largely independently in our dataset. We will include this analysis and the corresponding values in the revised manuscript.

**7. The authors seem to refer to the new Extended Fig. 1g; right?!**

We apologize for this oversight. We intended to refer to the bottom panel of Extended Data Fig. 1g, which shows the log–log transformation of the MSD curves. We have double-checked the manuscript and confirm that the

figure is cited correctly there; the error occurred only in the rebuttal letter, where the figure reference was mistyped.

**9. I think that the VVAC results for the LD population are interesting and worth showing to support the conclusions of the manuscript and to readers interested in molecular diffusion. Indeed, these results show strong evidence of confinement as a prime mechanism (i.e., long-term / "long-range constraints on motion") since VVAC is one of the most revealing statistical tests for distinguishing (sub)diffusion mechanisms [e.g., Golan et al, Nature Communications volume 8, Article number: 15851 (2017)]**

We thank the reviewer for highlighting the relevance of VVAC analysis for distinguishing diffusion mechanisms. We agree that the VVAC results for the LD population provide additional support for confinement-driven behavior, particularly at longer time scales where repeated interactions with nanodomains can impose long-range constraints on motion and reduce apparent mobility (reflected by lower α values). We included these analyses in the rebuttal for clarity and would be happy to incorporate the VVAC plots into the revised manuscript, together with appropriate references to the relevant literature.

Dear Dr. Casadio,

We are very pleased that the reviewers found our revisions responsive and that Reviewer 1 noted that the major concerns have been adequately addressed.

We appreciate the remaining minor suggestions from Reviewer 1 and have addressed them fully. These points primarily involve clarifications and the inclusion of additional statistical analyses and visualizations that we have already performed. Specifically, we have included:

- Appropriate statistical analyses and $P$ values for Extended Data Fig. 1i and Extended Data Fig. 6d
- VVACF plots, now included as Extended Data Fig. 1j

Please find our detailed point-by-point responses attached.

Thank you again for your time and consideration, and we look forward to hearing from you.

Arda, Bob, Tobi

2. I strongly recommend showing colocalization analysis of the results in Extended Data Fig. 6D to support the colocalization of the freely diffusive molecules with the ER marker. How does this colocalization change for the LD-localized trajectories?
We thank the reviewer for this suggestion. Colocalization analysis with ER and LD markers shows that 96 ± 1.5% of freely diffusive trajectories (N = 975) localize to ER regions, with only 4 ± 1.5% observed on LDs. In contrast, 70 ± 1.2% of confined trajectories (N = 502) localize to LDs, whereas 29 ± 1.2% colocalize with the ER. Importantly, when restricting the analysis to high-confidence trajectories, we do not observe tracks that fall outside either the ER or LD masks, indicating that the detected molecules are robustly associated with these membrane compartments. These results quantitatively support the visual overlay shown in Extended Data Fig. 6d and are consistent with the interpretation that freely diffusive molecules predominantly track along ER membranes, while confined trajectories are enriched on LDs. We included this colocalization analysis and the associated statistical summary in the figure legend.

3. Extended Data Fig 2c, 2d seem to relate to HSD17B13. Do the authors relate to other panels in the figure (e, f, or g), or is there some other mistake in labelling?
We have incorporated the GPAT4 related data into the main manuscript (Fig. 1f,g), while the corresponding HSD17B13 data were presented in Extended Data Fig. 2c,d. We chose to highlight GPAT4 in the main figure for clarity and simplicity, as the results for both proteins are highly consistent.

4. Extended Fig. 1i – please calculate and mention a correlation measure (e.g., Pearson's) to show that Dapp and alpha have no meaningful correlation.
We thank the reviewer for this suggestion. We performed a Pearson correlation analysis between the apparent diffusion coefficient ($D_{app}$) and the anomalous exponent ($\alpha$). This analysis revealed no significant relationship between the two variables (GPAT4: $r = -0.09$, $P = 0.15$; HSD17B13: $r = -0.03$, $P = 0.53$). These results indicate that the apparent diffusion coefficient and anomalous exponent vary largely independently in our dataset. We included this analysis and the corresponding values in the figure legend.

7. The authors seem to refer to the new Extended Fig. 1g; right?!
We apologize for this oversight. We intended to refer to the bottom panel of Extended Data Fig. 1g, which shows the log–log transformation of the MSD curves. We have double-checked the manuscript and confirm

that the figure is cited correctly there; the error occurred only in the rebuttal letter, where the figure reference was mistyped.

9. I think that the VVAC results for the LD population are interesting and worth showing to support the conclusions of the manuscript and to readers interested in molecular diffusion. Indeed, these results show strong evidence of confinement as a prime mechanism (i.e., long-term / "long-range constraints on motion") since VVAC is one of the most revealing statistical tests for distinguishing (sub)diffusion mechanisms [e.g., Golan et al, Nature Communications volume 8, Article number: 15851 (2017)]
We thank the reviewer for highlighting the relevance of VVAC analysis for distinguishing diffusion mechanisms. We agree that the VVAC results for the LD population provide additional support for confinement-driven behavior, particularly at longer time scales where repeated interactions with nanodomains can impose long-range constraints on motion and reduce apparent mobility (reflected by lower $\alpha$ values). We included the results of these analyses in Extended Data Fig 1j, together with appropriate references to the relevant literature.
